# Expression profiling of receptor tyrosine kinases in high-grade neuroendocrine carcinoma of the lung: a comparative analysis with adenocarcinoma and squamous cell carcinoma

**DOI:** 10.1007/s00432-015-1989-z

**Published:** 2015-05-20

**Authors:** Yuki Matsumura, Shigeki Umemura, Genichiro Ishii, Koji Tsuta, Shingo Matsumoto, Keiju Aokage, Tomoyuki Hishida, Junji Yoshida, Yuichiro Ohe, Hiroyuki Suzuki, Atsushi Ochiai, Koichi Goto, Kanji Nagai, Katsuya Tsuchihara

**Affiliations:** 1 Exploratory Oncology Research and Clinical Trial Center, National Cancer Center, Chiba, Japan; 2 Division of Thoracic Surgery, National Cancer Center Hospital East, Kashiwa, Japan; 3 Division of Thoracic Oncology, National Cancer Center Hospital East, Kashiwa, Chiba Japan; 4 Pathology Division, Research Center for Innovative Oncology, National Cancer Center Hospital East, Kashiwa, Japan; 5 Pathology Division, National Cancer Center Hospital, Tokyo, Japan; 6 Division of Thoracic Oncology, National Cancer Center Hospital, Kashiwa, Japan; 7 Department of Chest Surgery, Fukushima Medical University, Fukushima, Japan

**Keywords:** High-grade neuroendocrine carcinoma, Receptor tyrosine kinase, Immunohistochemical staining, Lung cancer

## Abstract

**Background:**

As the comprehensive genomic analysis of small cell lung cancer (SCLC) progresses, novel treatments for this disease need to be explored. With attention to the direct connection between the receptor tyrosine kinases (RTKs) of tumor cells and the pharmacological effects of specific inhibitors, we systematically assessed the RTK expressions of high-grade neuroendocrine carcinomas of the lung [HGNECs, including SCLC and large cell neuroendocrine carcinoma (LCNEC)].

**Patients and methods:**

Fifty-one LCNEC and 61 SCLC patients who underwent surgical resection were enrolled in this research. As a control group, 202 patients with adenocarcinomas (ADCs) and 122 patients with squamous cell carcinomas (SQCCs) were also analyzed. All the tumors were stained with antibodies for 10 RTKs: c-Kit, EGFR, IGF1R, KDR, ERBB2, FGFR1, c-Met, ALK, RET, and ROS1.

**Results:**

The LCNEC and SCLC patients exhibited similar clinicopathological characteristics. The IHC scores for each RTK were almost equivalent between the LCNEC and SCLC groups, but they were significantly different from those of the ADC or SQCC groups. In particular, c-Kit was the only RTK that was remarkably expressed in both LCNECs and SCLCs. On the other hand, about 20 % of the HGNEC tumors exhibited strongly positive RTK expression, and this rate was similar to those for the ADC and SQCC tumors. Intriguingly, strongly positive RTKs were almost mutually exclusive in individual tumors.

**Conclusions:**

Compared with ADC or SQCC, LCNEC and SCLC had similar expression profiles for the major RTKs. The exclusive c-Kit positivity observed among HGNECs suggests that c-Kit might be a distinctive RTK in HGNEC.

**Electronic supplementary material:**

The online version of this article (doi:10.1007/s00432-015-1989-z) contains supplementary material, which is available to authorized users.

## Introduction

Large cell neuroendocrine carcinoma (LCNEC) is distinguished from small cell lung carcinoma (SCLC) based on its histological criteria, that is, a larger cell size, abundant cytoplasm, prominent nucleoli, vesicular nuclei or coarse chromatin, and a polygonal rather than a fusiform shape (Battafarano et al. 2005). Despite these differences, LCNEC and SCLC share many similarities in terms of not only immunohistochemistry, but also clinical characteristics (Gupta et al. 2004; Asamura et al. 2006; Fernandez and Battafarano 2006; Gollard et al. 2010; Nakachi et al. 2010; Dobashi et al. 2011; Li et al. 2012; Peifer et al. 2012; Rudin et al. 2012; Sun et al. 2012). Consequently, these lesions are often grouped together as high-grade neuroendocrine carcinoma (HGNEC). LCNEC also shares genetic alterations that are commonly seen in SCLCs, such as *TP53*, *RB1*, and *EP300* (Jones et al. 2004; Peifer et al. 2012; Rudin et al. 2012; CLCGP-NGM 2013), suggesting a genetic similarity to SCLC. However, little is known about the differences in the protein expression profiles between these two histological types.

In addition, only fragmented information on therapeutically relevant gene alterations is available for HGNECs. Two reports regarding integrative genomic analyses of SCLC have shown that transcriptional deregulation (for example, via *RB1*, *SOX2*, and *MYC* family members and chromatin modifiers) might have a role in its biology.(Peifer et al. 2012; Rudin et al. 2012) To date, however, attempts to develop targeted therapies for these transcriptional deregulations have had limited success. Recently, we performed whole-exome sequencing of 51 Asian SCLC patients and demonstrated that the SCLC genome possessed distinguishable genetic features in the PI3K/AKT/mTOR pathway (Umemura et al. 2014). In this report, both gene mutations and copy number variations were analyzed, and genetic alterations in various targetable well-known receptor tyrosine kinase (RTK) genes were detected, but these variations were not correlated with the genetic changes in the PI3K/AKT/mTOR pathway, and their functional roles have remained unclear.

As already known, RTKs are the initial signaling gate on the cell membrane. Given their pivotal roles in tumor initiation and progression, RTKs have become one of the most prominent target families for drug development (IASLC 2009; Umemura et al. 2014). Therefore, in the present study, we analyzed the protein expressions of the major RTKs of the HGNEC tumors, which we examined using whole-exome sequencing, and compared them with those of adenocarcinoma (ADC) and squamous cell carcinoma (SQCC) to identify biologically distinctive alterations in HGNECs.

## Materials and methods

### Patient selection

Between 1992 and 2012, a total of 51 consecutive LCNEC and 61 consecutive SCLC patients underwent surgical resections in National Cancer Center Hospital East, Japan; these patients were enrolled in the present study. As a control group, 202 adenocarcinoma (ADC) and 122 squamous cell carcinoma (SQCC) patients who underwent surgery between 2010 and 2012 were also analyzed. We obtained the clinicopathological data of all the enrolled patients from our database and analyzed the results.

### Histological studies

The surgical specimens had been fixed in 10 % formalin or 100 % methyl alcohol. The specimens were sliced through the largest diameter of the primary tumor, and all the sections were embedded in paraffin. Serial 4-μm sections were stained using the hematoxylin and eosin (HE) method, the Alcian blue-periodic acid-Schiff (AB-PAS) method for the detection of cytoplasmic mucin production, or the Elastica van Gieson (EVG) or the Victoria-blue van Gieson (VVG) method for the detection of elastic fibers. All the histological materials included in this series were reviewed by two pathologists (Y.M. and G.I.). The pathological stage was determined based on the 7th edition TNM classification of the International Union Against Cancer (UICC) (IASLC 2009). We diagnosed the histological types of lung cancer according to The World Health Organization (WHO) classification of lung cancer (Travis et al. 2004). LCNEC was distinguished from SCLC based on this classification. Morphologically, LCNEC is characterized by neuroendocrine morphologic features (organoid nesting, trabecular growth, and rosette-like and palisading patterns), large tumor cells (three times larger in diameter than a small resting lymphocyte) with a low nuclear/cytoplasmic ratio, numerous nucleoli, a high mitotic rate (>10 in 10 high-power fields), and a large degree of necrosis. The neuroendocrine features were confirmed for all LCNEC and SCLC patients by the presence of at least one positive neuroendocrine marker, such as chromogranin, synaptophysin, or CD56.

### Antibodies and immunohistochemical staining for RTK

Tissue microarray (TMA) slides of all the enrolled tumors were used for immunohistochemical staining (IHC). We retrieved two cores with diameters of 2 mm from a selected paraffin block from each tumor and utilized them for the TMA. The companies from which we purchased the antibodies and the detailed IHC procedures are shown in Supplemental Table 1. The antibodies targeted the following 10 RTKs: c-Kit, EGFR, insulin-like growth factor 1 receptor (IGF1R), kinase insert domain receptor (KDR), human epidermal growth factor receptor 2 (ERBB2), fibroblast growth factor receptor 1 (FGFR1), c-MET, anaplastic lymphoma kinase (ALK), RET, and ROS1. The reason that we chose these 10 RTKs in this research was because they had been widely reported, and their IHC tests were also well validated in our laboratory. Immunostaining was performed on 4-μm paraffin-embedded tissue sections. The slides were deparaffinized in xylene and dehydrated in a graded ethanol series, and endogenous peroxidase was blocked with 3 % hydrogen peroxide in absolute methyl alcohol. After epitope retrieval, the slides were washed with phosphate-buffered saline and incubated overnight with the primary antibodies. For the secondary antibodies, the slides were subsequently incubated with EnVision™ (Dako, Glostrup, Denmark) for 30 min at room temperature followed by the color reaction developed in 2 % 3, 3′-diaminobenzidine in 50 mM Tris buffer (pH 7.6) containing 0.3 % hydrogen peroxidase. The reaction products were stained with diaminobenzidine (DAB) and were counterstained with hematoxylin. ERBB2 and c-MET were stained using the Ventana Ultraview DAB detection kit in Ventana Benchmark XT stainer (Ventana Medical Systems, Tucson, AZ, USA). The IHC staining of ALK and ROS1 was performed according to previous reports (Nitta et al. 2013; Yoshida et al. 2013a).

### Immunohistochemical scoring

**Fig. 1:**
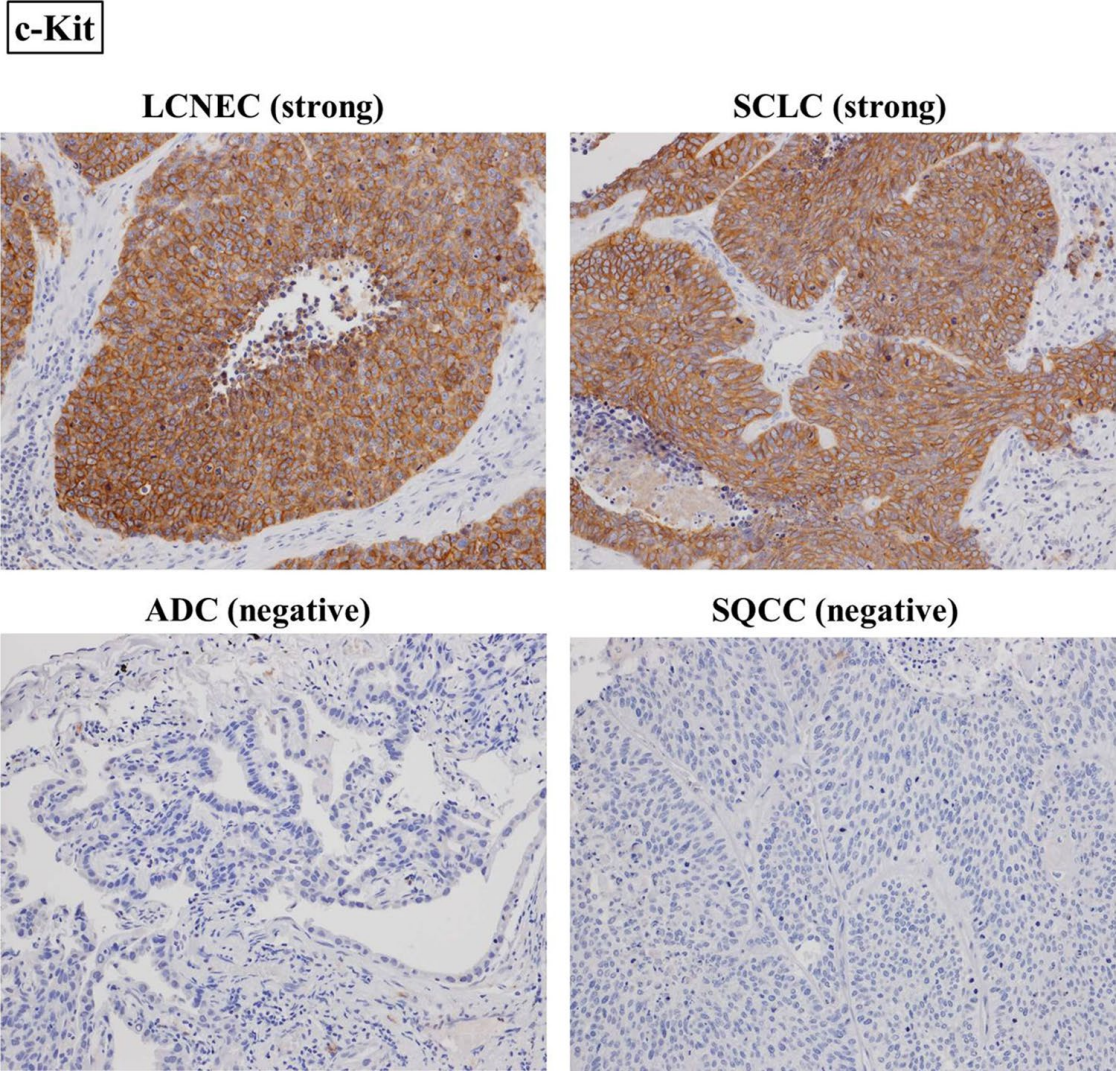
Immunohistochemical staining of LCNEC, SCLC, ADC, and SQCC. LCNEC and SCLC were strongly stained for c-Kit, while ADC and SQCC were negative. IGF1R was weakly positive in LCNEC and SCLC and strongly positive in SQCC. ADC was negative for IGF1R. On the other hand, c-Met was negative in LCNEC and SCLC and strongly positive in ADC. SQCC was weakly positive for c-Met

All the stained tissue sections were semiquantitatively scored and evaluated independently under a light microscope by two pathologists (Y.M. and G.I.) who had no knowledge of the patients’ clinicopathological data. The labeling scores for the tumor cells were calculated by multiplying the percentage of positive tumor cells in each lesion (0–100 %) by the staining intensity level (0 = negative, 1 = weak, 2 = strong). When the scores differed, the pathologists re-examined the slides together, discussed the results, and reached an agreement. In this research, tumors with an IHC score of 20–90 were defined as “weakly positive,” since the median score of each RTK in each histological type was 20 or less. On the other hand, tumors with an IHC score of 100 or more were defined as “strongly positive,” because such tumors belonged to the top 10 % of highest scores for each RTK (Fig. 1 and Supplemental Figure 1).

### Statistical analysis

The Mann–Whitney *U* test or the Fisher’s exact test was used to test differences between two groups for statistical significance. All the reported *P* values were two-sided, and the significance level was set at <0.05. Overall survival and recurrence-free survival were calculated using the Kaplan–Meier method, and significance was tested using a log-rank test. The analyses were performed using the SPSS 11.0 statistical software program (Dr. SPSS II for Windows, standard version 11.0; SPSS Inc., Chicago, IL, USA).

## Results

### Clinicopathological features of LCNEC and SCLC

The clinicopathological characteristics of the LCNEC, SCLC, ADC, and SQCC patients are shown in Table 1. Of the enrolled patients, 43 (84 %), 50 (82 %), 120 (59 %), and 112 (92 %) of the patients with LCNEC, SCLC, ADC, and SQCC were men, respectively. Smoking history was observed in 50 (98 %), 59 (98 %), 128 (64 %), and 121 (99 %) of the patients with LCNEC, SCLC, ADC, and SQCC, respectively. No significant differences in the clinicopathological characteristics of the patients with LCNEC and those with SCLC were seen. Compared with the ADC group, significantly more men and smokers were observed in the LCNEC and SCLC group (all *P* < 0.01). As for the pStage, no significant differences were observed among the four types of lung cancers. Vascular invasion and lymphatic permeation were significantly more frequent in patients with LCNEC or SCLC than in patients with ADC (*P* < 0.01).

**Table 1:** Clinicopathological characteristics of LCNEC and SCLC, compared with those of adenocarcinoma and squamous cell carcinoma

	LCNEC	SCLC	ADC	SQCC
	*n* = 51	*n* = 61	*n* = 202	*n* = 122
Gender: men (%)	43 (84)	50 (82)	120 (59)	112 (92)
Age: median (range)	69 (53–84)	67 (22–86)	68 (40–93)	70 (42–85)
Smoking: ever (%)	50 (98)	59 (97)	128 (64)	121 (99)
Pack-year ≥50	26 (51)	24 (39)	46 (23)	71 (58)
pStage I	31 (62)	34 (56)	131 (65)	47 (39)
II	10 (19)	14 (23)	36 (18)	44 (36)
III	10 (19)	11 (18)	31 (15)	31 (25)
IV	0	2 (3)	4 (2)	0
Pleural invasion	20 (39)	19 (31)	77 (38)	56 (41)
Vascular invasion	39 (76)	52 (85)	84 (42)	83 (68)
Lymphatic permeation	18 (35)	23 (38)	31 (15)	28 (23)

*P* value	LCNEC versus SCLC	LCNEC versus ADC	LCNEC versus SQCC	SCLC versus ADC	SCLC versus SQCC
Men	0.80	**<0.01**	0.15	**<0.01**	**0.05**
Age	0.84	0.52	0.56	0.36	0.06
Smoking	1.00	**<0.01**	0.54	**<0.01**	0.21
PY ≥ 50	0.25	**<0.01**	0.38	**0.01**	0.02
pStage I–II versus III–IV	0.94	0.71	0.41	0.48	0.54
Pleural invasion	0.43	0.90	0.42	0.39	0.08
Vascular invasion	0.33	**<0.01**	0.26	**<0.01**	**0.01**
Lymphatic permeation	0.85	**<0.01**	0.10	**<0.01**	**0.04**

Statistically significant values are indicated in bold (*p* ≤ 0.05)

LCNEC and SCLC: from 1992 to 2011

ADC and SQCC: from 2010 to 2012

Chi-square test

### Immunohistochemical staining scores for each histological type

**Fig. 2:**
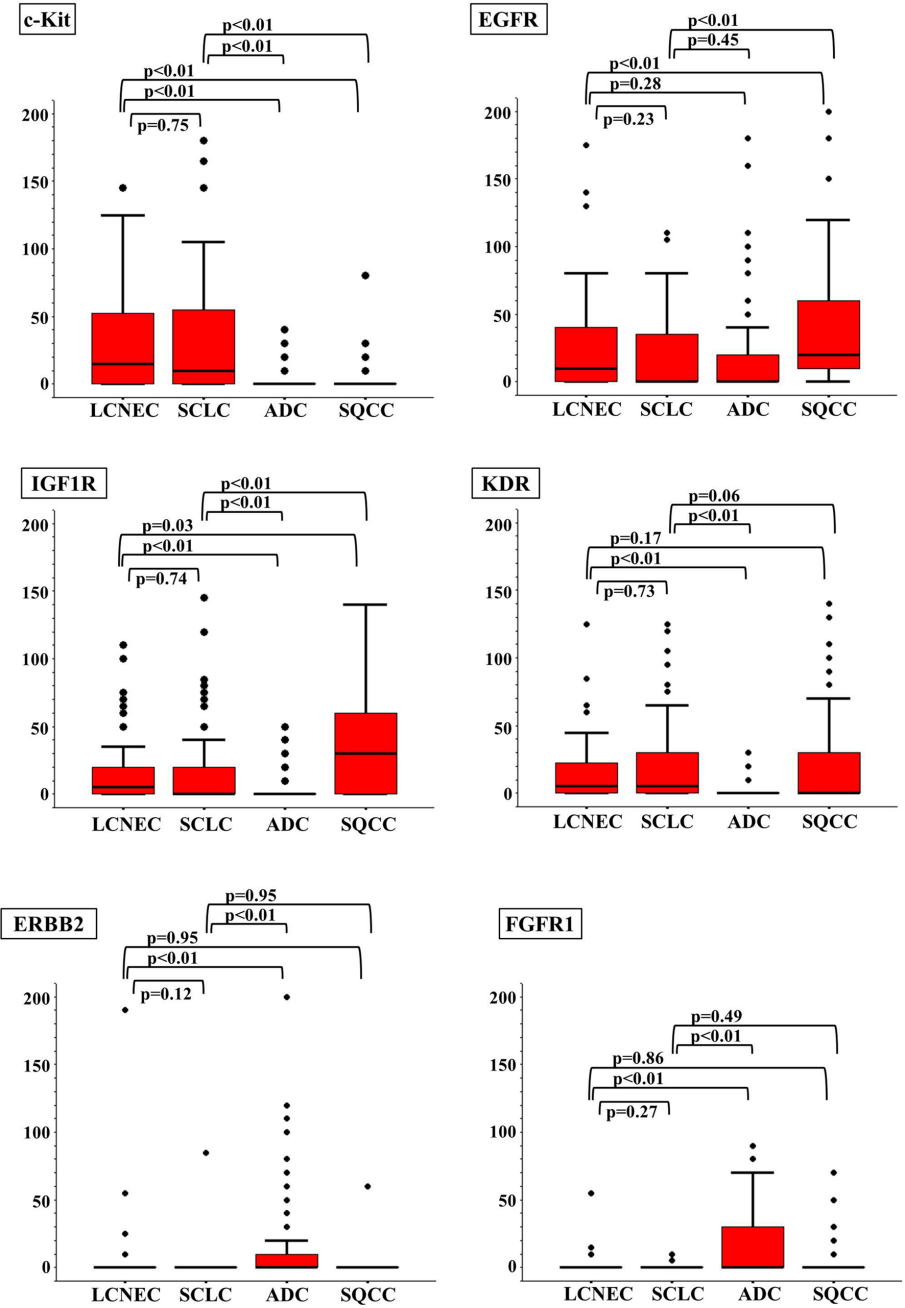

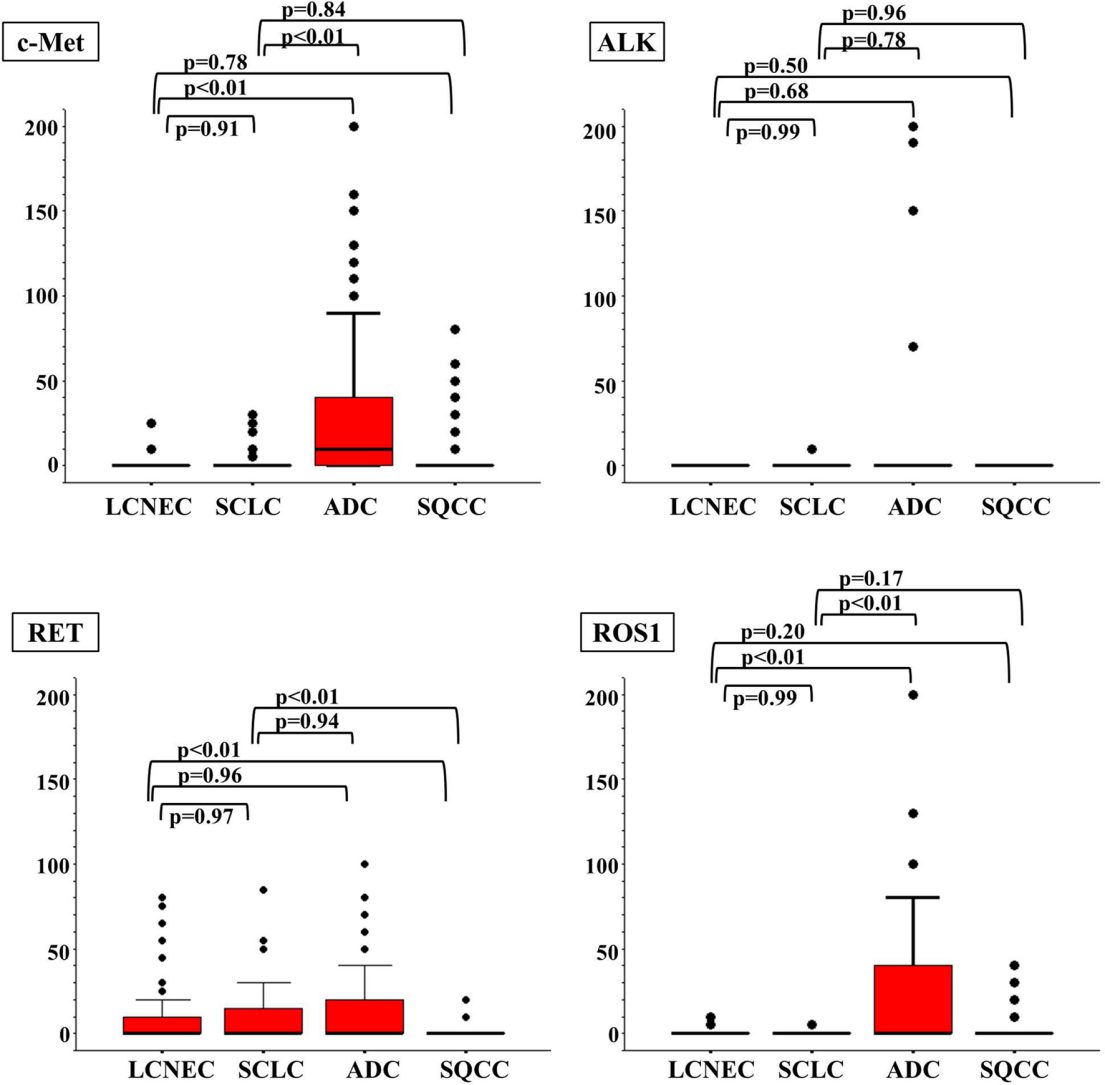
Immunohistochemical staining scores for each histological type. Compared with ADC, both LCNEC and SCLC had significantly higher scores for c-Kit, IGF1R, and KDR and lower scores for ERBB2, FGFR1, c-Met, and ROS1. Compared with SQCC, they had significantly higher scores for c-Kit, KDR, and RET and lower scores for EGFR and IGF1R. Therefore, c-Kit was the only RTK that was remarkably expressed in LCNEC and SCLC, compared with both ADC and SQCC

Figure 2 and Table 2 show the immunohistochemical staining score (IHC score) for each RTK (c-Kit, EGFR, IGF1R, KDR, ERBB2, FGFR1, c-Met, ALK, RET, and ROS1) in each histological type. Three points of interest were identified from these scores. First, no significant differences in the IHC scores for each RTK were observed between the LCNEC and the SCLC tumors. Second, the IHC scores for the LCNEC and SCLC tumors were significantly different from those for the ADC and SQCC tumors. Specifically, both the LCNEC and the SCLC tumors had significantly higher scores for c-Kit, IGF1R, and KDR and lower scores for ERBB2, FGFR1, c-Met, and ROS1, compared with the ADC tumors. When compared with the SQCC tumors, the LCNEC and the SCLC tumors had significantly higher scores for c-Kit, KDR, and RET and lower scores for EGFR and IGF1R. Thirdly, c-Kit was the only RTK that was remarkably expressed in LCNEC and SCLC tumors, compared with both ADC and SQCC tumors.

**Table 2:** Immunohistochemical staining score due to histological type

RTKs	LCNEC (*n* = 51) Mean	SCLC (*n* = 61) Mean	Adc (*N* = 202) Mean	Sqcc (*N* = 122) Mean
c-Kit	31.7 ± 5.3	32.2 ± 5.2	0.9 ± 0.7	1.6 ± 1.6
EGFR	26.4 ± 10.9	20.6 ± 7.7	15.9 ± 3.9	44.3 ± 8.7
IGF1R	18.2 ± 7.6	17.7 ± 8.1	2.7 ± 1.1	33.5 ± 6.5
KDR	18.1 ± 7.7	22.5 ± 8.2	0.7 ± 0.6	19.4 ± 6.0
ERBB2	5.5 ± 7.8	1.4 ± 2.8	10.0 ± 3.6	0.5 ± 1.0
FGFR1	1.8 ± 2.2	0.2 ± 0.4	19.1 ± 4.0	1.7 ± 1.5
c-Met	1.1 ± 8.3	2.0 ± 7.6	28.6 ± 4.1	5.9 ± 5.4
ALK	0	0.2 ± 3.9	3.0 ± 3.1	0
RET	10.5 ± 5.5	8.7 ± 4.0	13.2 ± 3.1	0.8 ± 0.6
ROS1	0.3 ± 0.4	0.2 ± 0.2	23.6 ± 4.7	2.3 ± 1.4

*P* value	LCNEC versus SCLC	LCNEC versus ADC	LCNEC versus SQCC	SCLC versus ADC	SCLC versus SQCC
c-Kit	0.75	**<0.01**	**<0.01**	**<0.01**	**<0.01**
EGFR	0.23	0.28	**<0.01**	0.45	**<0.01**
IGF1R	0.74	**<0.01**	**0.03**	**<0.01**	**<0.01**
KDR	0.73	**<0.01**	0.17	**<0.01**	0.06
ERBB2	0.12	**<0.01**	0.95	**<0.01**	0.95
FGFR1	0.27	**<0.01**	0.86	**<0.01**	0.49
c-Met	0.91	**<0.01**	0.78	**<0.01**	0.84
ALK	0.99	0.68	0.50	0.78	0.96
RET	0.97	0.96	**<0.01**	0.94	**<0.01**
ROS1	0.99	**<0.01**	0.20	**<0.01**	0.17

Statistically significant values are indicated in bold (*p* ≤ 0.05)

Mann–Whitney’s *U* test

### RTK expressions in individual patients according to each histological type

**Fig. 3:**
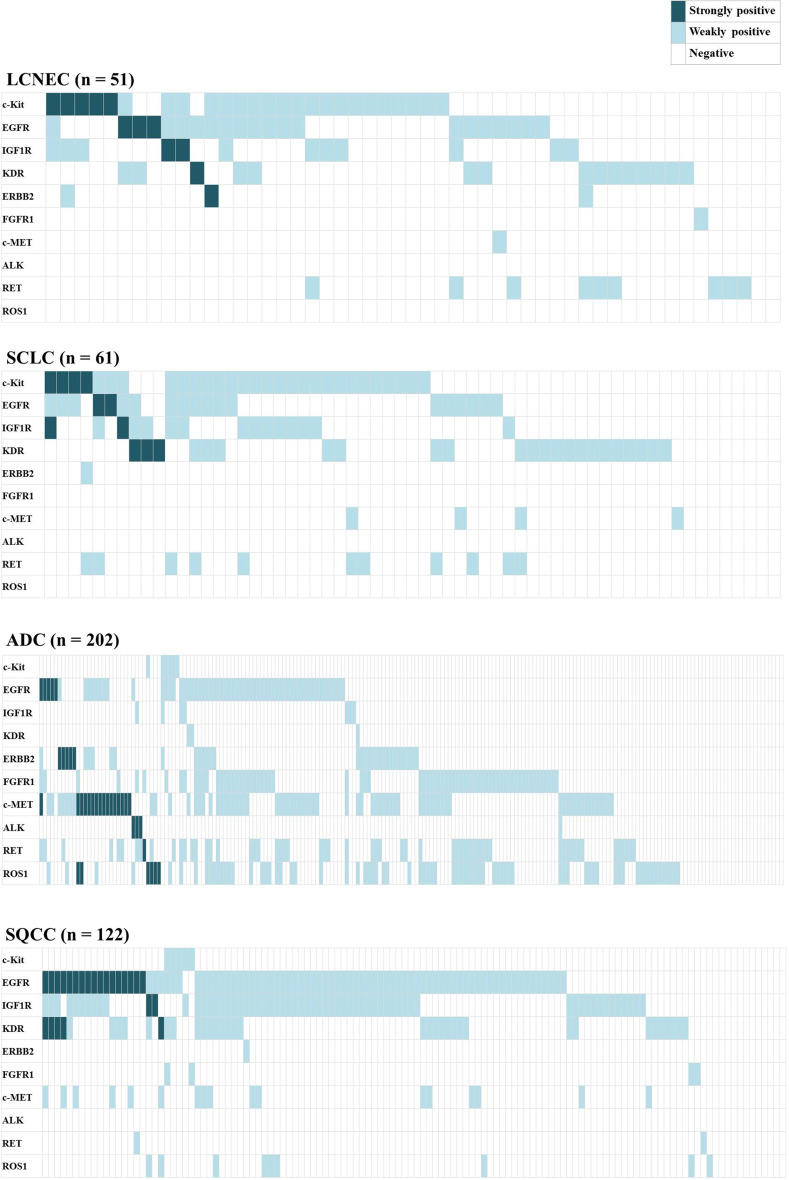
Distribution of RTK positivity in individual patients according to each histological type. Dark blue indicates a strongly positive RTK (IHC score of 100 or more), light blue indicates a weakly positive RTK (IHC score of between 20 and 90), and white indicates RTK negativity (IHC score of 0 or 10). More than 80 % of the tumors in each histological type had at least one strongly or weakly positive RTK. The distributions of positive RTKs were similar in LCNEC and SCLC but varied from those of ADC and SQCC. Regarding the strongly positive RTKs, the LCNEC group had a total of 12 (24 %) tumors with some type of strongly positive RTK. The SCLC group had 10 (16 %), the ADC group had 33 (16 %), and the SQCC had 20 (16 %) strongly positive RTKs. The strongly positive RTKs were almost mutually exclusive in individual tumors

We also analyzed the RTK expressions in individual patients and plotted each RTK score for individual cases in Fig. 3. “Strongly positive (dark blue in Fig. 3),” “weakly positive (light blue),” and “negative” in this figure were determined according to the criteria described in the “Methods” section. This figure and Supplemental Table 2A revealed that more than 80 % of the tumors in each histological type had at least one strongly or weakly positive RTK. The number of tumors with two or more positive RTKs was 28 (55 %) for the LCNEC tumors, 23 (56 %) for the SCLC tumors, 131 (65 %) for the ADC tumors, and 82 (67 %) for the SQCC tumors. The numbers of LCNEC and SCLC tumors with two or more positive RTKs were nearly identical (*P* = 0.93), but the numbers tended to be lower for LCNEC and SCLC tumors than for ADC or SQCC tumors (Supplemental Table 2B). Supplemental Table 3 shows the number of patients with positive RTKs according to each histological type. These numbers were similar between the LCNEC and SCLC tumors for all the RTKs, but the numbers for the LCNEC and SCLC tumors differed significantly from those for the ADC and SQCC tumors for some RTKs. Compared with the ADC tumors, the frequency of positive RTKs among the LCNEC and SCLC tumors was significantly higher for c-Kit (LCNEC: 49 %, SCLC: 47 %, and ADC: 3 %), IGF1R (LCNEC: 31 %, SCLC: 28 %, and ADC: 5 %), and KDR (LCNEC: 29 %, SCLC: 38 %, and ADC: 1 %) and lower for ERBB2 (LCNEC: 6 %, SCLC: 2 %, and ADC: 17 %), FGFR1 (LCNEC: 2 %, SCLC: 0 %, and ADC: 35 %), c-Met (LCNEC: 2 %, SCLC: 6 %, and ADC: 43 %), and ROS1 (LCNEC: 0 %, SCLC: 0 %, and ADC: 40 %). Compared with SQCC, on the other hand, the frequency of positive RTKs among the LCNEC and SCLC tumors was significantly higher for c-Kit (LCNEC: 49 %, SCLC: 47 %, and SQCC: 4 %) and lower for EGFR (LCNEC: 41 %, SCLC: 31 %, and SQCC: 69 %), IGF1R (LCNEC: 31 %, SCLC: 28 %, and SQCC: 62 %), and c-Met (LCNEC: 2 %, SCLC: 6 %, and SQCC: 14 %). These tables and Fig. 3 revealed that the distributions of positive RTKs were similar for the LCNEC and SCLC tumors but differed from those for the ADC and SQCC tumors.

Regarding the strongly positive RTKs, the LCNEC group exhibited strongly positive c-Kit in five tumors, EGFR in three, IGF1R in two, and KDR and ERBB2 in one each; thus, a total of 12 (24 %) LCNEC tumors had some kind of strongly positive RTK. The SCLC group had strongly positive c-Kit in four tumors, EGFR in two, IGF1R in two, and KDR in three, for a total of 10 (16 %) SCLC tumors with a strongly positive RTK (one tumor was strongly positive for both c-Kit and IGF1R). On the other hand, the ADC group exhibited strongly positive EGFR in five tumors, ERBB2 in five, c-Met in 16, ALK in three, RET in one, and ROS1 in six, for a total of 33 (16 %) ADC tumors with a strongly positive RTK. One ADC tumor was positive for both EGFR and c-Met, and two tumors were positive for both c-Met and ROS1. The SQCC group exhibited strongly positive EGFR in 17 tumors, IGF1R in two, and KDR in five, for a total of 20 (16 %) SQCC tumors with a strongly positive RTK. Four tumors were positive for both EGFR and KDR, but none of the tumors had three or more strongly positive RTKs among any of the histological types. Of interest, Fig. 3 shows that each tumor with a strongly expressed RTK was almost mutually exclusive.

### RTK expression and genomic alterations

Supplemental Figure 2 shows the relationship between the *EGFR* mutation status and RTK expression in ADC. The *EGFR* mutation status was assessed using the PCR invader assay for 147 patients. Of them, 58 (39 %) harbored *EGFR* mutations: 40 had point mutations in exon 21 (L858R), 16 had exon 19 deletions, and 2 had minor mutations. Three of the four (75 %) cases with strongly positive EGFR expression also harbored *EGFR* mutations, while 20 of the 21 (95 %) cases with strongly positive non-EGFR-RTK expression did not have *EGFR* mutations. As for SQCC, the *EGFR* mutation status was examined in 25 of the 122 (20 %) enrolled patients, but *EGFR* mutations were not identified in any of the cases.

On the other hand, Supplemental Figure 3 shows the relationship between RTK expression and genomic alterations in SCLC. The genomic alteration data for these SCLC tumors were extracted from our previous report (Umemura et al. 2014). Minimal correlations were observed between a strong positivity for RTK expression and genomic alterations. However, one patient with strong c-Kit expression had an in-frame deletion and a copy number gain in the *KIT* gene.

### Overall survival of patients with LCNEC or SCLC

The overall survival (OS) rates of the LCNEC and SCLC groups are shown in Supplemental Figure 4. The median follow-up period was 61 months. The 3-year OS rates for the LCNEC patients and for the SCLC patients were both 66 %. No significant difference in the OS was seen between the LCNEC group and the SCLC group (*P* = 0.53). When LCNEC and SCLC were grouped together and considered as HGNEC, the 3-year OS of the patients with HGNEC tumors with a strongly positive RTK (*n* = 22) and that of patients with those without it (*n* = 90) was 70 and 63 %, respectively; these percentages were not significantly different (*P* = 0.17) (Supplemental Figure 5). The 3-year OS rates of patients with and those without c-Kit positivity were 69 % and 63 %, respectively, and these values were also not significantly different (*P* = 0.57) (Supplemental Figure 6).

## Discussion

We performed an extensive RTK expression study for all four major histological types of lung cancer, focusing on HGNEC. Intriguingly, the overall RTK expression patterns of LCNEC and SCLC were similar, but they were quite different from those for ADC or SQCC. The similarity between LCNEC and SCLC has long been described since the concept of LCNEC was first proposed in the 1990s (Travis et al. 1991, 1998a, b). Based on the morphological features of LCNEC and SCLC, many reports have suggested a similarity in their clinical behaviors (Asamura et al. 2006; Kinoshita et al. 2013) and sensitivities to chemotherapy (Le Treut et al. 2013; Niho et al. 2013). Jones et al. (2004) also reported that they could not distinguish LCNEC from SCLC based on gene expression profiling. From this aspect, our analysis of the protein expression levels of major RTKs supports the past consensus.

In the present research, we analyzed the RTK expression levels in individual patients and plotted each RTK score for individual cases, since diversity in the expression levels was observed in individual cases even among those with the same histological type. This analysis revealed that about 20 % of LCNEC and SCLC tumors had some kind of strongly stained RTK, such as c-Kit, EGFR, IGF1R, KDR, or ERBB2. Interestingly, most of these strongly staining RTKs were mutually exclusive, evoking the oncogenic driver mutations (*EGFR*, *KRAS*, *ALK*, and *ROS1*) observed in lung adenocarcinoma. Additionally, we examined the relationship between the *EGFR* mutation status and RTK expression in ADC and showed that strongly positive non-EGFR–RTKs tended to be mutually exclusive with *EGFR* mutations. We anticipate that this exclusiveness might reflect the importance of these highly expressed RTKs for tumor proliferation, survival, or invasiveness.

In this study, one strongly positive ERBB2 tumor existed in the LCNEC group, but no strongly positive ERBB2 tumors were seen in the SCLC group. According to a recent report from The Clinical Lung Cancer Genome Project (CLCGP) and Network Genomic Medicine (NGM) (CLCGP-NGM 2013), a total of two (5 %) LCNEC tumors with ERBB2 amplification, but no SCLC tumors, have been reported, consistent with the present findings. The identification of LCNEC tumors with high ERBB2 expression levels suggests that a subset of these tumors might be sensitive to ERBB2 inhibitors, similar to ERBB2-positive gastric cancer and breast cancer (Asaoka et al. 2011; Stern 2012; Kumler et al. 2014).

Some LCNEC and SCLC tumors have been reported to exhibit FGFR1 amplification (Peifer et al. 2012); however, no LCNEC or SCLC tumors that were strongly positive for FGFR1 were observed in our study. Although the amplification of FGFR1 is reportedly predominant in squamous cell carcinomas, the association with overexpression was inconclusive (Pros et al. 2013). Further examination of FGFR1 alterations is needed.

The histological type-specific findings in the present study were in accordance with previous reports of a high frequency of c-Kit expression in HGNEC (Pelosi et al. 2004a, b, Dy et al. 2005; Schneider et al. 2010; Lu et al. 2012), EGFR expression in SQCC (Mountzios et al. 2010; Pirker et al. 2012; Jiang et al. 2013), and ALK expression in a minority (three of 202 tumors, 1.5 %) of ADC tumors (Chen et al. 2012; Park et al. 2012; Nitta et al. 2013). Of note, the expression of c-Kit was considerably higher in HGNEC than in ADC or SQCC, suggesting its biological importance for tumorigenesis in HGNEC. High expression levels of c-Kit in LCNEC have also been previously reported (Araki et al. 2003; Rossi et al. 2003; Casali et al. 2004; Pelosi et al. 2004a, b, Rossi et al. 2005; Lopez-Martin et al. 2007; Schneider et al. 2010; Lu et al. 2012). Similar to our findings, Rossi et al. (2003) reported that c-KIT was frequently expressed in both SCLC and LCNEC, but not in ADC or SQCC. However, two phase II studies using imatinib, a c-Kit inhibitor, failed to demonstrate any clinical benefit even among selected SCLC patients harboring c-Kit-expression (Dy et al. 2005; Schneider et al. 2010). Unlike the situation for gastrointestinal stromal tumors (GIST), activating mutations in the *KIT* gene were minimally associated with the immunohistochemical expression of c-Kit in HGNEC. Actually, Rossi et al. reported that c-Kit was strongly expressed in 52 of the 83 LCNECs (62.7 %), but no activating mutation was detected in the *KIT* gene. We combined the current results with our previous data for whole-exon sequencing and a copy number analysis of SCLC samples (Umemura et al. 2014) and observed a similar tendency. Nevertheless, one SCLC case with strong c-Kit expression also had a mutation and amplification of the *KIT* gene. This rare case might be a candidate for targeted therapy, and IHC for c-Kit might be useful for patient screening. On the other hand, in imatinib-resistant GIST, the PI3K/AKT/mTOR pathway is a major contributor to proliferation and survival (Floris et al. 2013). This pathway has also been proposed as an actionable signaling cascade that is active in SCLC (Arriola et al. 2008; Ilic et al. 2011). This finding implies that a combined treatment with PI3K/AKT/mTOR inhibitor might be potentially effective for increasing the sensitivity to c-Kit inhibitors in HGNEC. In addition to tyrosine kinase inhibitors, antibodies are expected to be effective for tumor cells overexpressing target RTKs. A novel treatment using anti-c-kit antibody has been attempted in vitro (Yoshida et al. 2013b). Further studies will require the clarification of specific biological features and the development of c-Kit-targeted therapies.

As for the survival of patients with LCNEC and those with SCLC, the OS rates for both histological types were not significantly different, similar to the results of previous reports (Asamura et al. 2006; Kinoshita et al. 2013). Furthermore, the OS curve of HGNEC patients with RTK positivity was not significantly different from those without RTK positivity (Supplemental Figs. 2 and 3). The reasons for the similarity in OS curves were thought to include the small population of strongly positive RTKs, the short follow-up periods, the contributions of other RTK, and driver oncogenes that were not assessed in the present study. We did not find any significant effect of c-Kit expression on survival, similar to the results of previous reports (Rossi et al. 2005; Lopez-Martin et al. 2007). However, Casali et al. (2004) reported that c-Kit expression in LCNEC was a negative prognostic factor. Further investigation of possible correlations in larger studies is warranted.

In conclusion, LCNEC and SCLC are relatively similar, compared with ADC and SQCC, even at the protein expression level. Based on this background, the development of molecular-targeted agents for SCLC and LCNEC could be combined into the development of treatments for “HGNEC” (Pelosi et al. 2004b; Schneider et al. 2010; Sun et al. 2012; CLCGP-NGM 2013). Although RTK positivity cannot be used as the sole criterion for targeted therapies for HGNEC, strongly positive RTKs were observed in a mutually exclusive manner, suggesting their biological importance for tumorigenesis in HGNEC. In the future, we plan to perform both expression and genomic analyses of RTK in non-resectable advanced HGNEC tumors, in addition to surgically resected samples.

## Electronic supplementary material

Below is the link to the electronic supplementary material.

Supplementary material 1 (DOCX 15 kb)

Supplementary material 2 (TIFF 25513 kb)

Supplementary material 3 (TIFF 25513 kb)

Supplementary material 4 (TIFF 25513 kb)

Supplementary material 5 (TIFF 25512 kb)

Supplementary material 6 (TIFF 25512 kb)

Supplementary material 7 (TIFF 25512 kb)

Supplementary material 8 (TIFF 25512 kb)

Supplementary material 9 (TIFF 25511 kb)

Supplementary material 10 (TIFF 25512 kb)

Supplementary material 11 (TIFF 25511 kb)

Supplementary material 12 (TIFF 25513 kb)

Supplementary material 13 (TIFF 25513 kb)

Supplementary material 14 (TIFF 8519 kb)

Supplementary material 15 (TIFF 8519 kb)

Supplementary material 16 (TIFF 8519 kb)

Supplementary material 17 (DOCX 33 kb)
