# Supplementary material for: Expression profiling of receptor tyrosine kinases in high-grade neuroendocrine carcinoma of the lung: a comparative analysis with adenocarcinoma and squamous cell carcinoma
Source: J Cancer Res Clin Oncol. 2015 May 20;141(12):2159–70. doi: 10.1007/s00432-015-1989-z (PMC4630254; doi:10.1007/s00432-015-1989-z)
Supplement: Supplementary file 1 — Supplementary material 1 (DOCX 15 kb) [file 432_2015_1989_MOESM1_ESM.docx]

Supplemental Figure 1: Immunohistochemical staining of LCNEC, SCLC, ADC, and SQCC. We stained all tumors with antibodies for 10 RTKs: c-Kit, EGFR, IGF1R, KDR, ERBB2, FGFR1, c-Met, ALK, RET and ROS1.

Supplemental Figure 2: Relationship between *EGFR* mutation status and IHC RTK expression in adenocarcinoma. *EGFR* mutation status was assessed in 147 patients. Of them, 58 (39%) harbored *EGFR* mutations: 40 had exon 21 (L858R), 16 had exon 19 deletions, and 2 had minor mutations. Three of 4 (75%) cases with strongly positive EGFR expression also harbored *EGFR* mutations, while 20 of 21 (95%) cases with strongly positive non-EGFR-RTK expression did not have *EGFR* mutation.

Supplemental Figure 3: Relationship between genomic alteration and IHC RTK expression in SCLC. There is little relevance between strong positivity of RTK expression and genomic alterations. However, there is one patient who had not only strongly expressed c-Kit, but also in-frame deletion and copy number gain of c-Kit.

Supplemental Figure 4: Overall survival (OS) curves for patients with LCNEC and SCLC. The 3-year OS rates for the LCNEC and SCLC patients were both 66%. No significant differences in OS were observed between the LCNEC group and the SCLC group (*p* = 0.53).

Supplemental Figure 5: Overall survival (OS) of patients with HGNEC with strongly positive RTKs. The 3-year OS rates of HGNEC patients with strongly positive RTKs (n = 22) and patients with other tumors (n = 90) were 70% and 63%, respectively; these values were not significantly different (*p* = 0.17).

Supplemental Figure 6: Overall survival (OS) of HGNEC patients with or without c-Kit positivity. The 3-year OS rates of patients with and those without c-Kit positivity were 69% and 63%, respectively; these values were not significantly different (*p* = 0.57).
