# Supplementary figures and images for: Expression profiling of receptor tyrosine kinases in high-grade neuroendocrine carcinoma of the lung: a comparative analysis with adenocarcinoma and squamous cell carcinoma
Source: J Cancer Res Clin Oncol. 2015 May 20;141(12):2159–70. doi: 10.1007/s00432-015-1989-z (PMC4630254; doi:10.1007/s00432-015-1989-z)

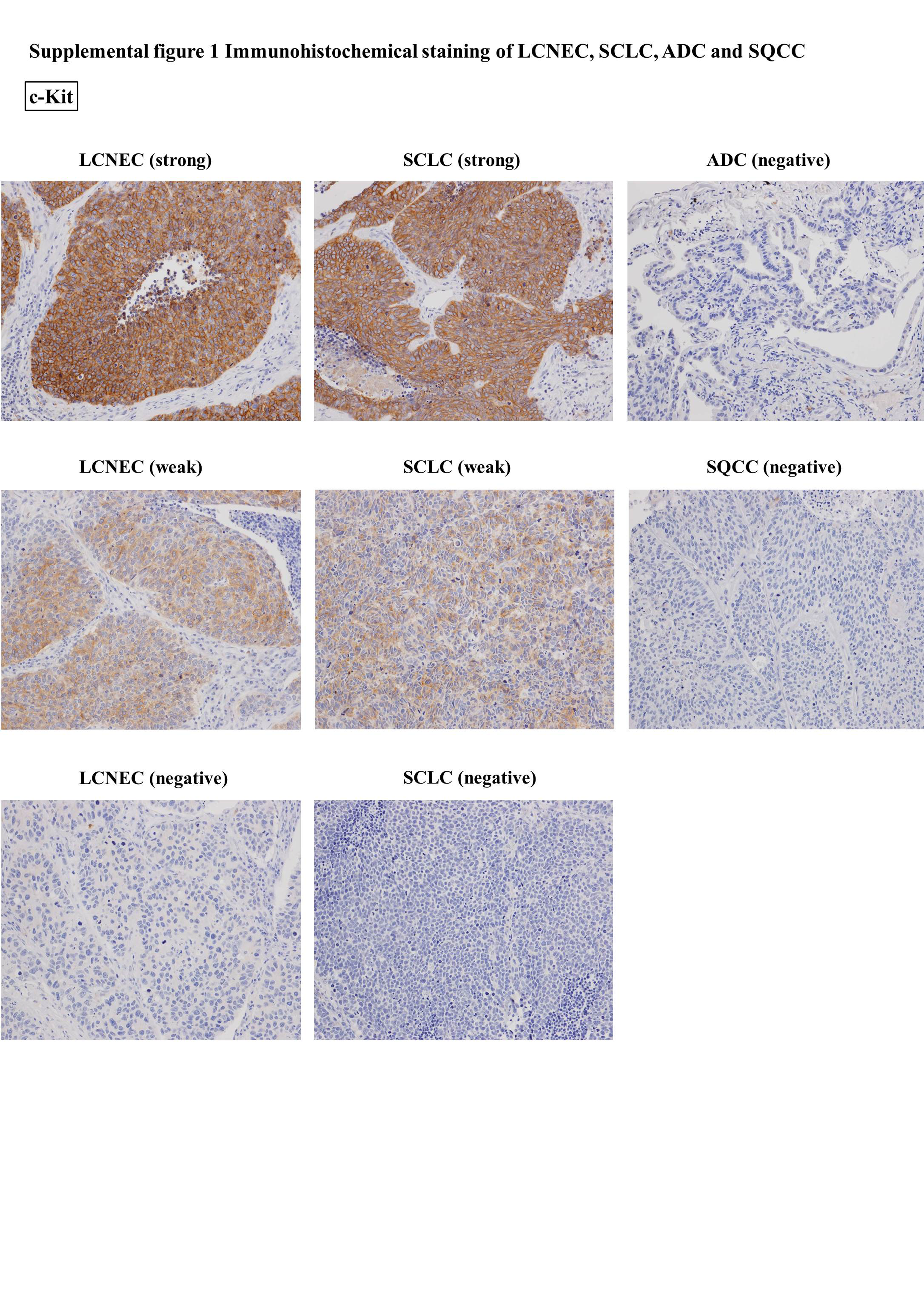

Supplement: Supplementary file 2 — Supplementary material 2 (TIFF 25513 kb) [file 432_2015_1989_MOESM2_ESM.tif]

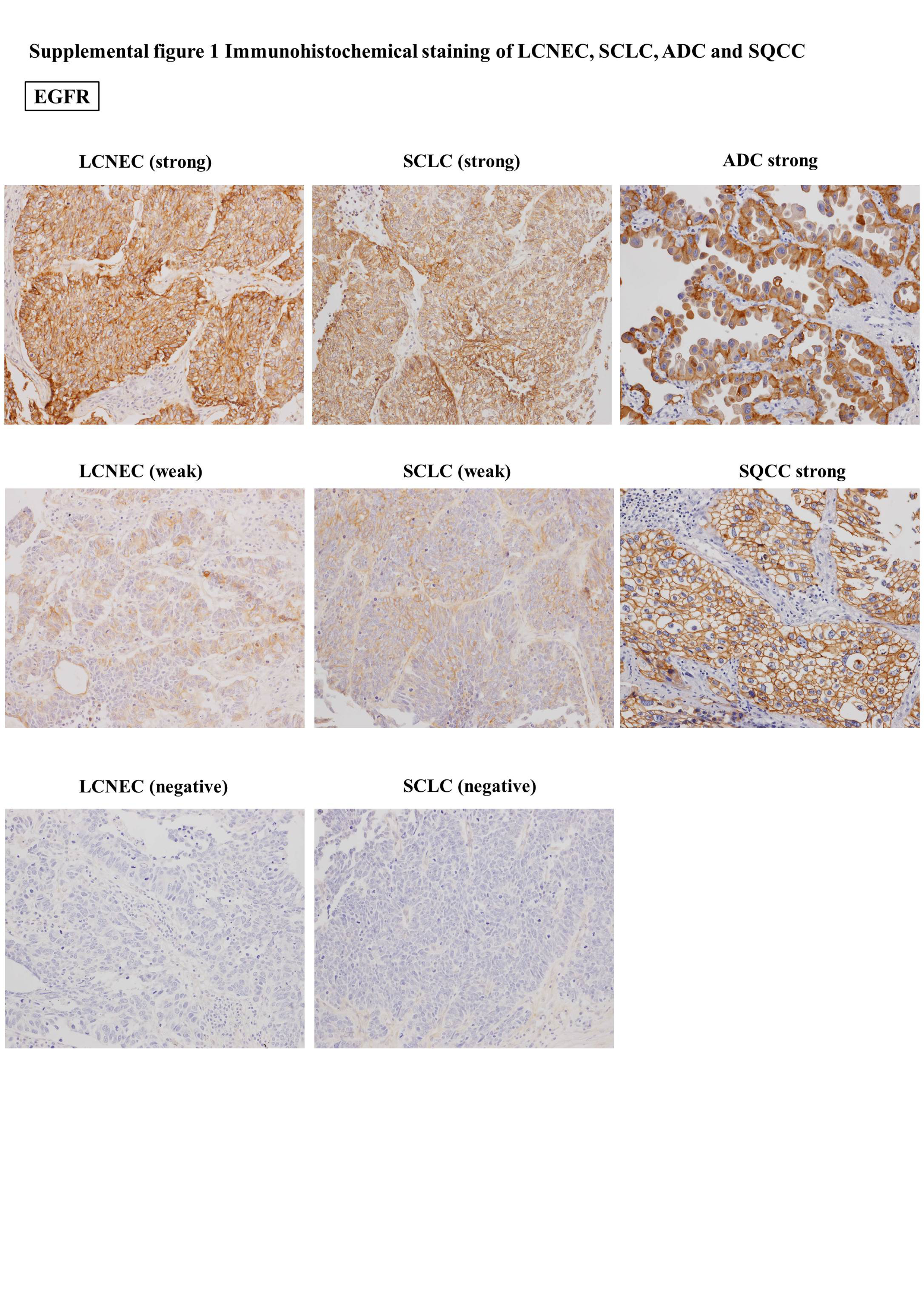

Supplement: Supplementary file 3 — Supplementary material 3 (TIFF 25513 kb) [file 432_2015_1989_MOESM3_ESM.tif]

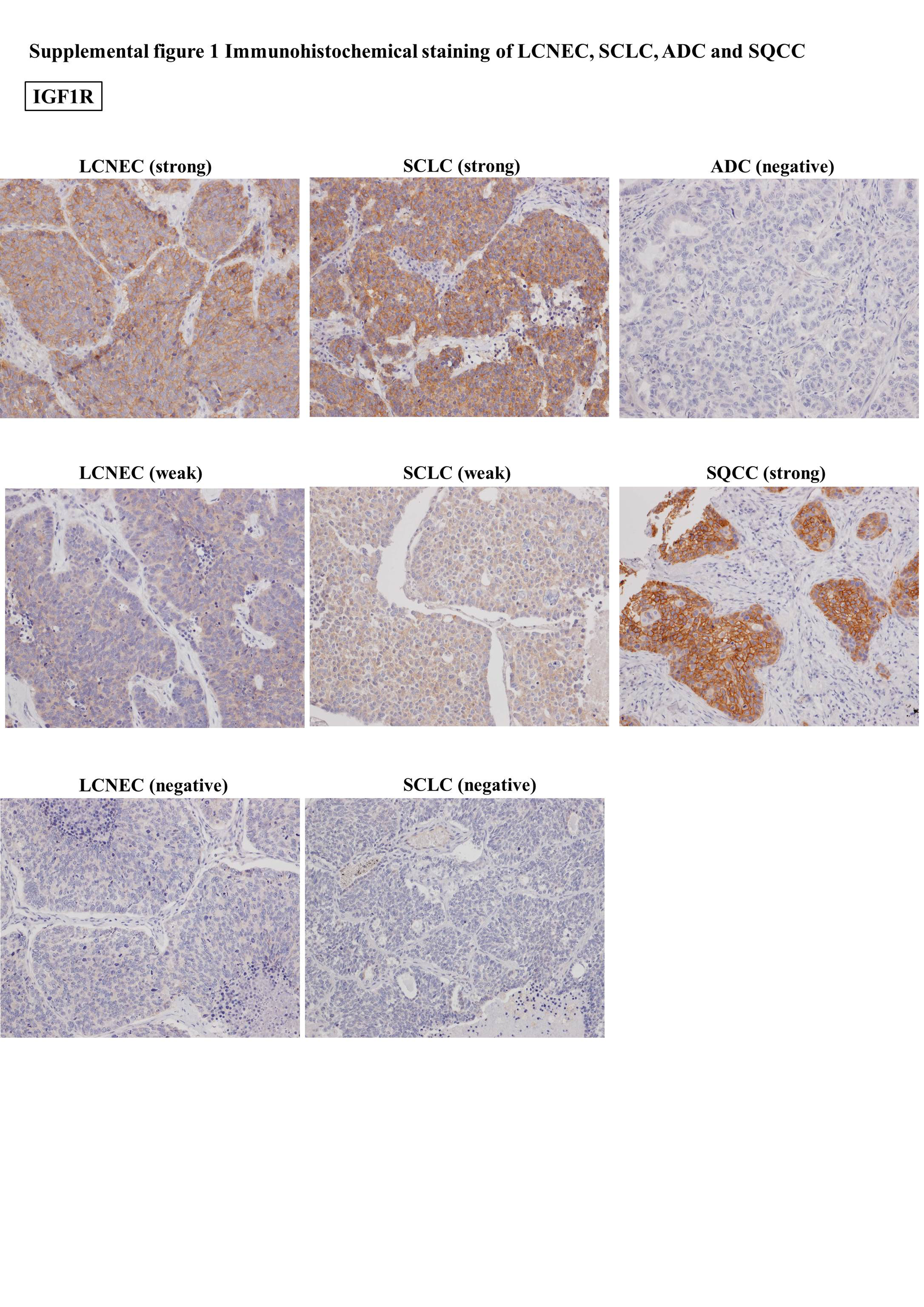

Supplement: Supplementary file 4 — Supplementary material 4 (TIFF 25513 kb) [file 432_2015_1989_MOESM4_ESM.tif]

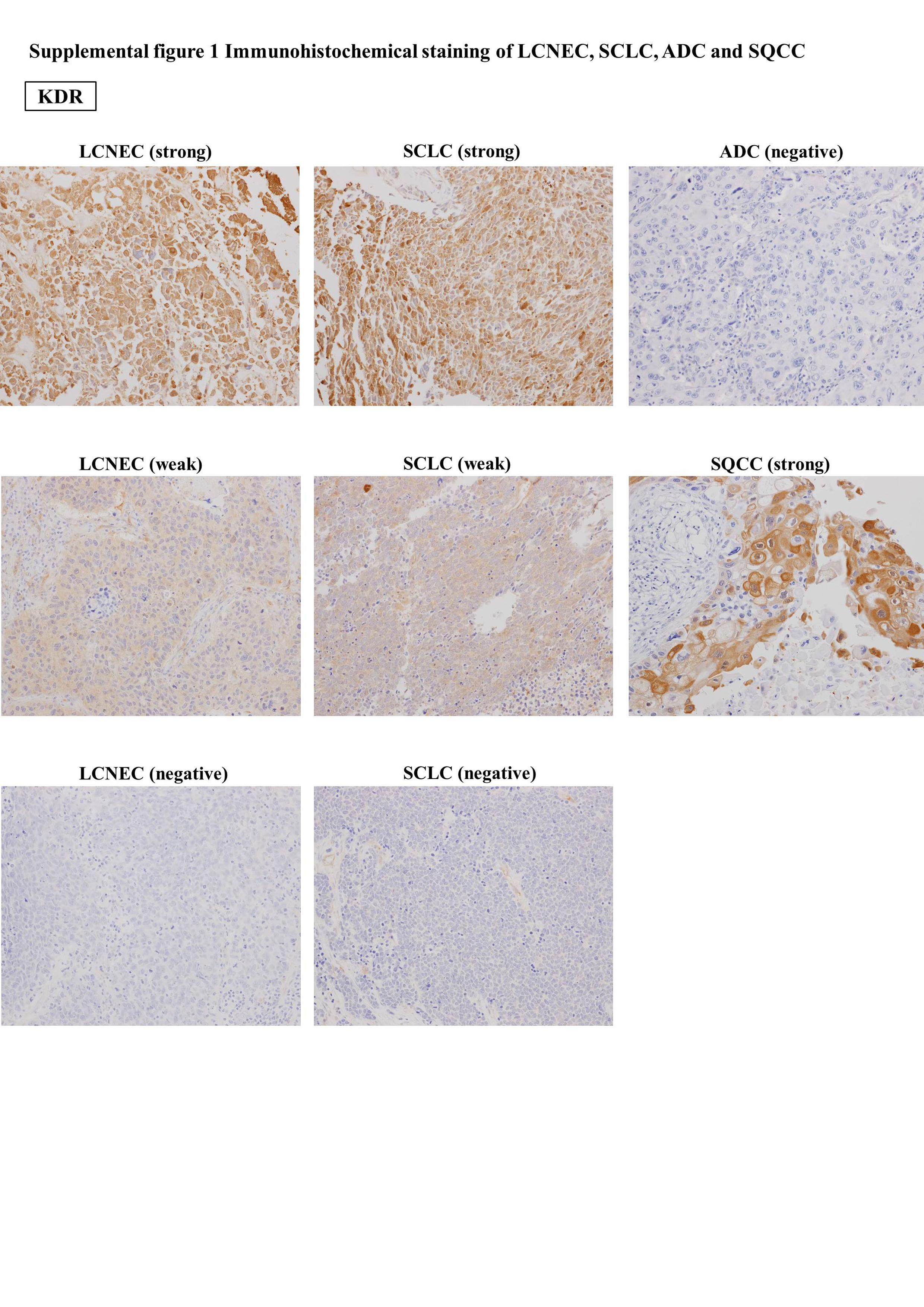

Supplement: Supplementary file 5 — Supplementary material 5 (TIFF 25512 kb) [file 432_2015_1989_MOESM5_ESM.tif]

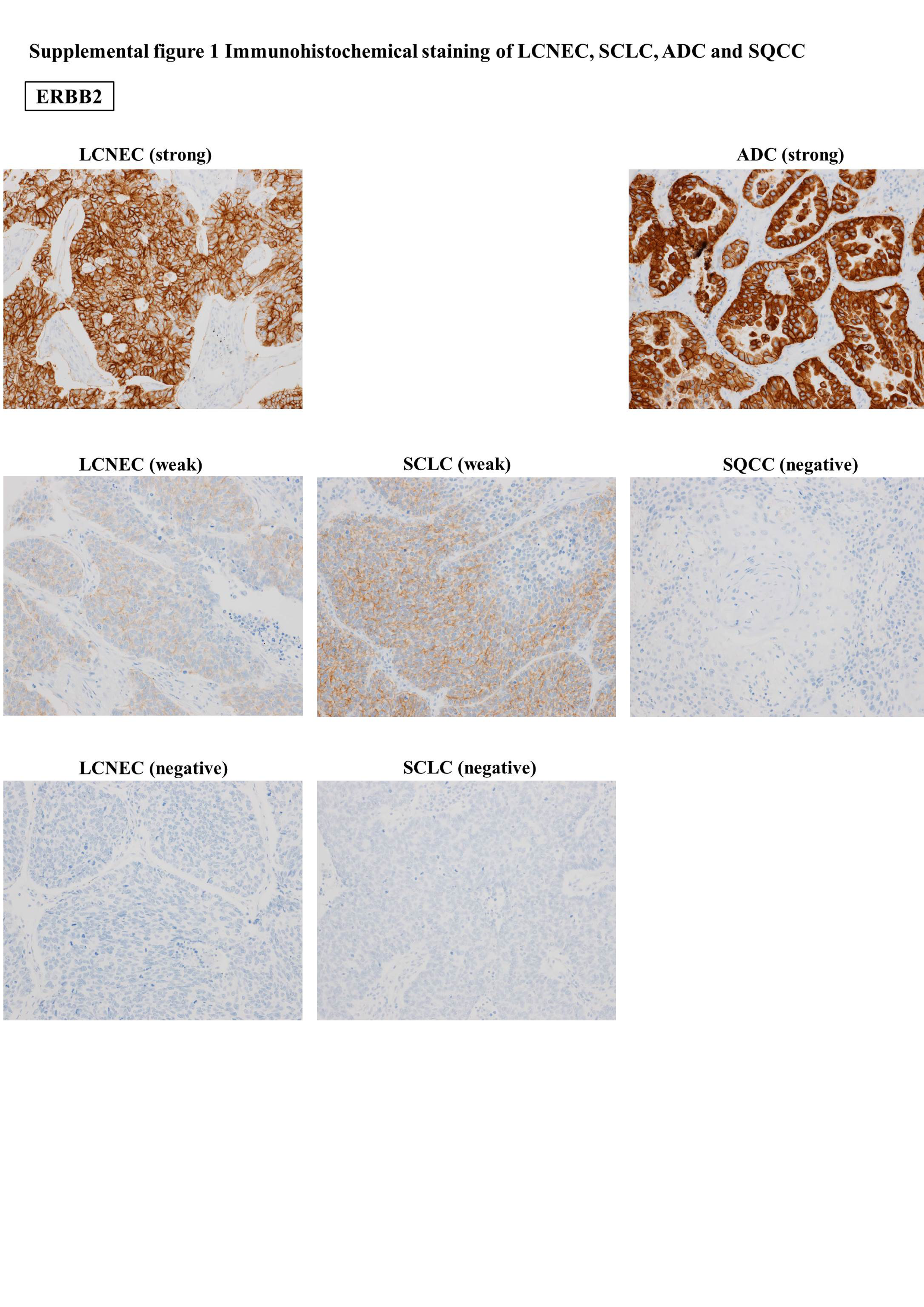

Supplement: Supplementary file 6 — Supplementary material 6 (TIFF 25512 kb) [file 432_2015_1989_MOESM6_ESM.tif]

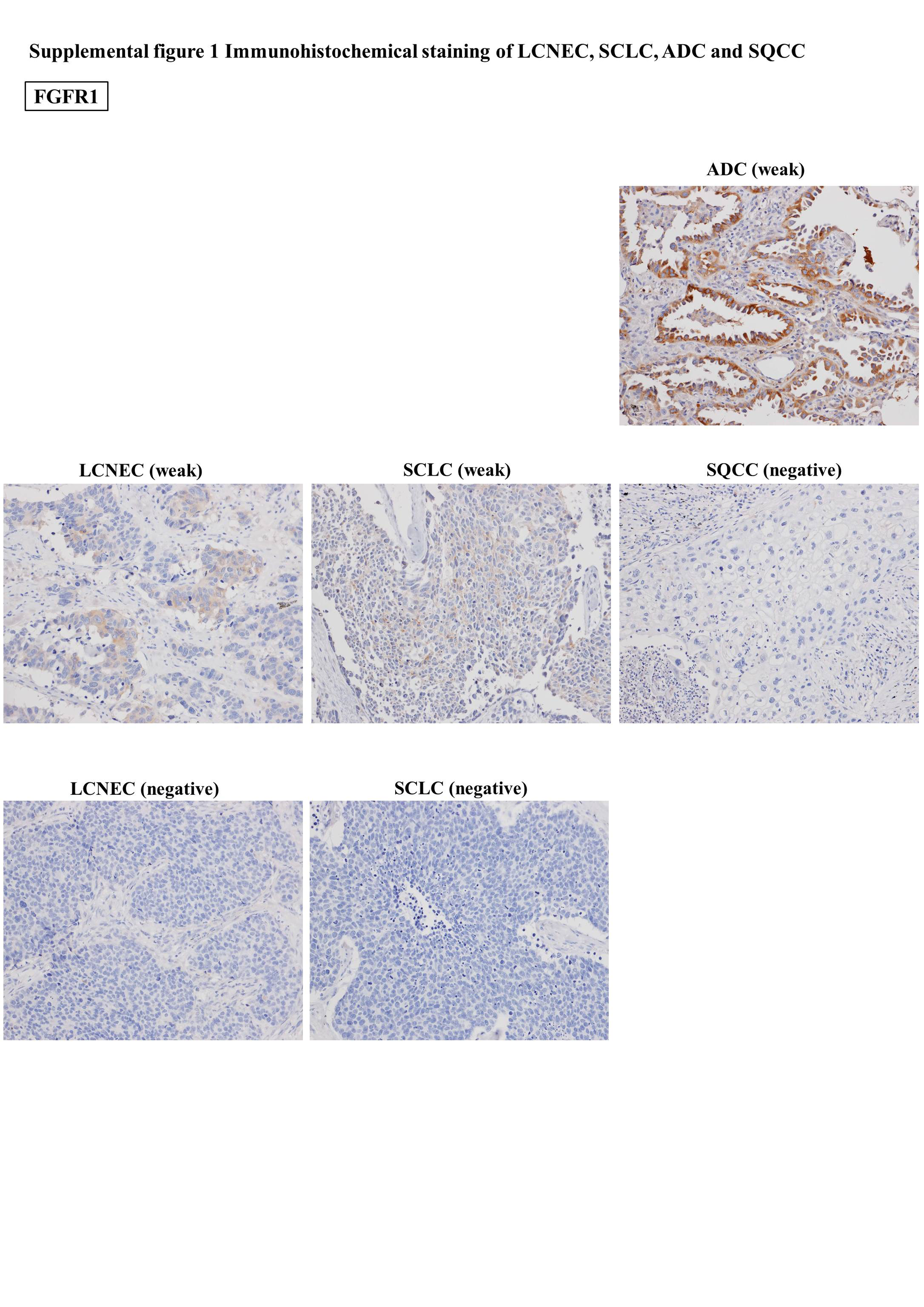

Supplement: Supplementary file 7 — Supplementary material 7 (TIFF 25512 kb) [file 432_2015_1989_MOESM7_ESM.tif]

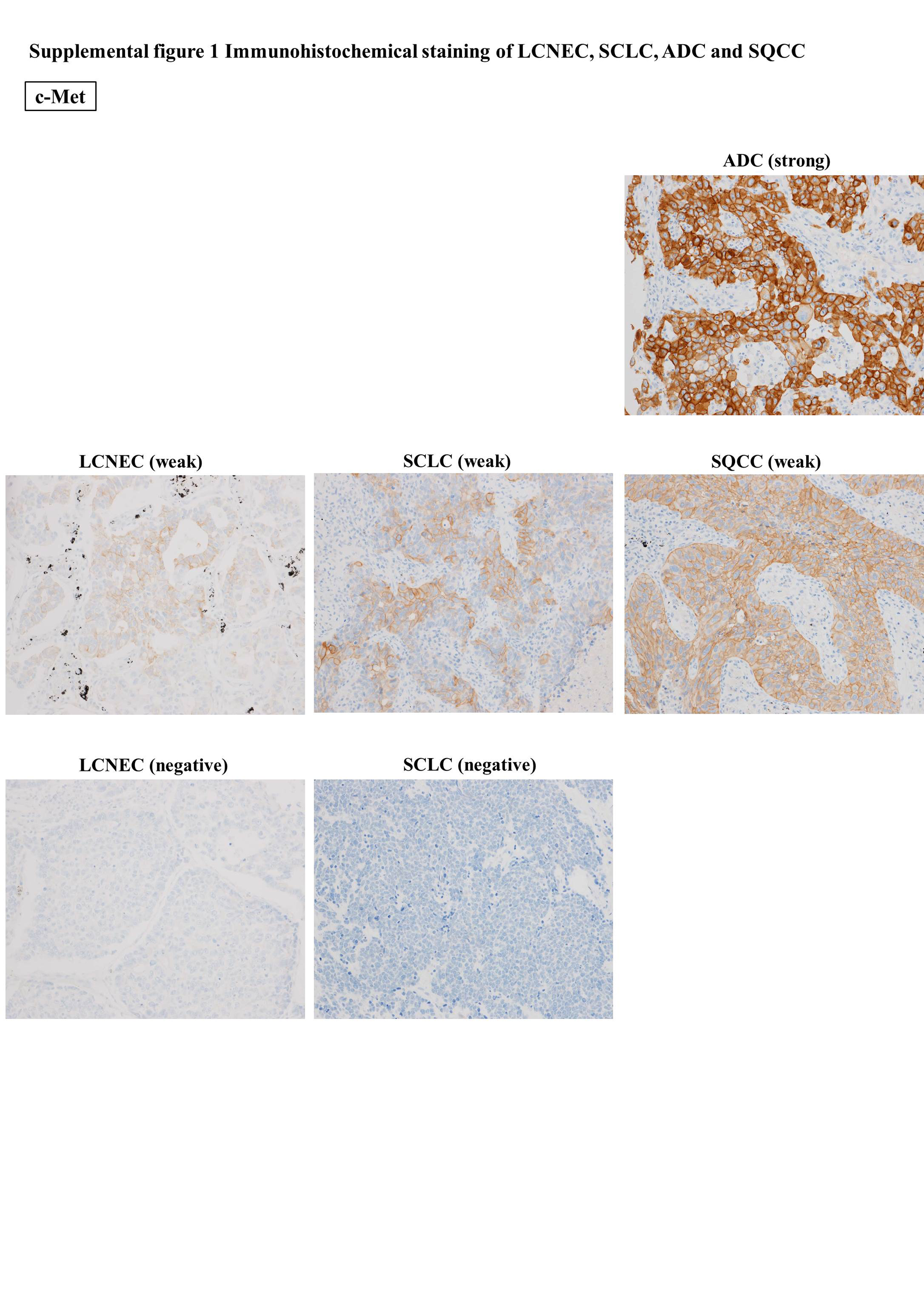

Supplement: Supplementary file 8 — Supplementary material 8 (TIFF 25512 kb) [file 432_2015_1989_MOESM8_ESM.tif]

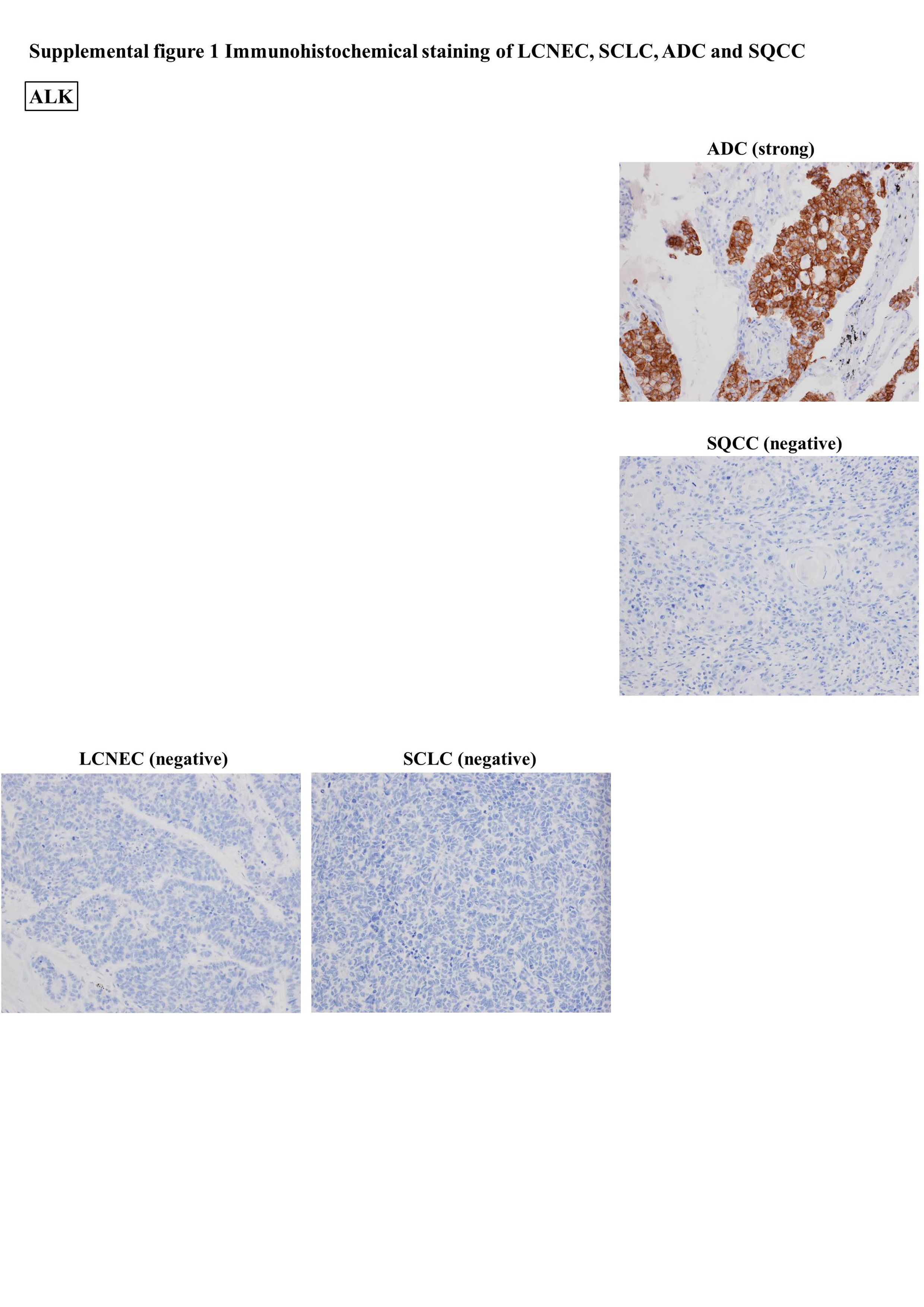

Supplement: Supplementary file 9 — Supplementary material 9 (TIFF 25511 kb) [file 432_2015_1989_MOESM9_ESM.tif]

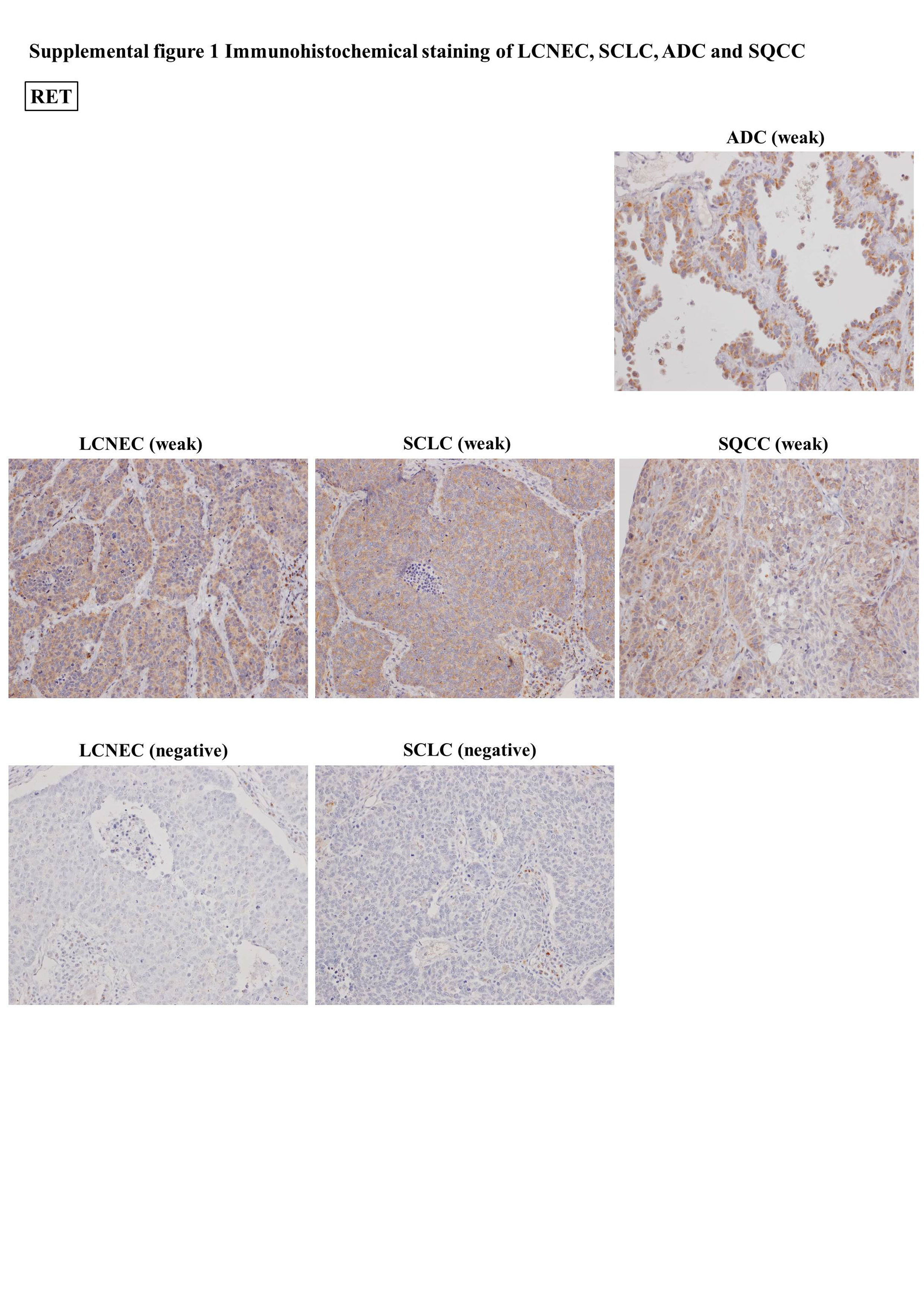

Supplement: Supplementary file 10 — Supplementary material 10 (TIFF 25512 kb) [file 432_2015_1989_MOESM10_ESM.tif]

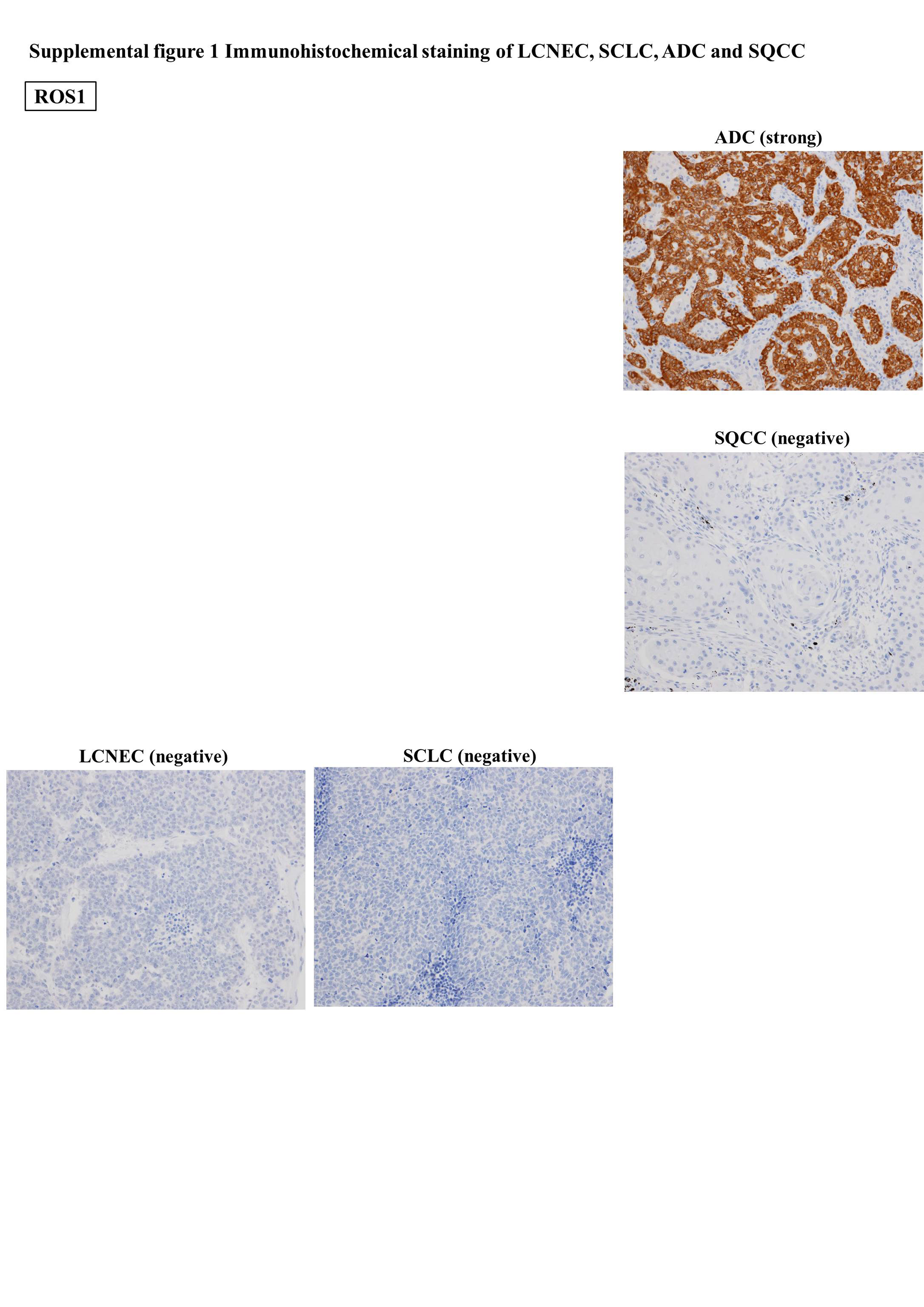

Supplement: Supplementary file 11 — Supplementary material 11 (TIFF 25511 kb) [file 432_2015_1989_MOESM11_ESM.tif]

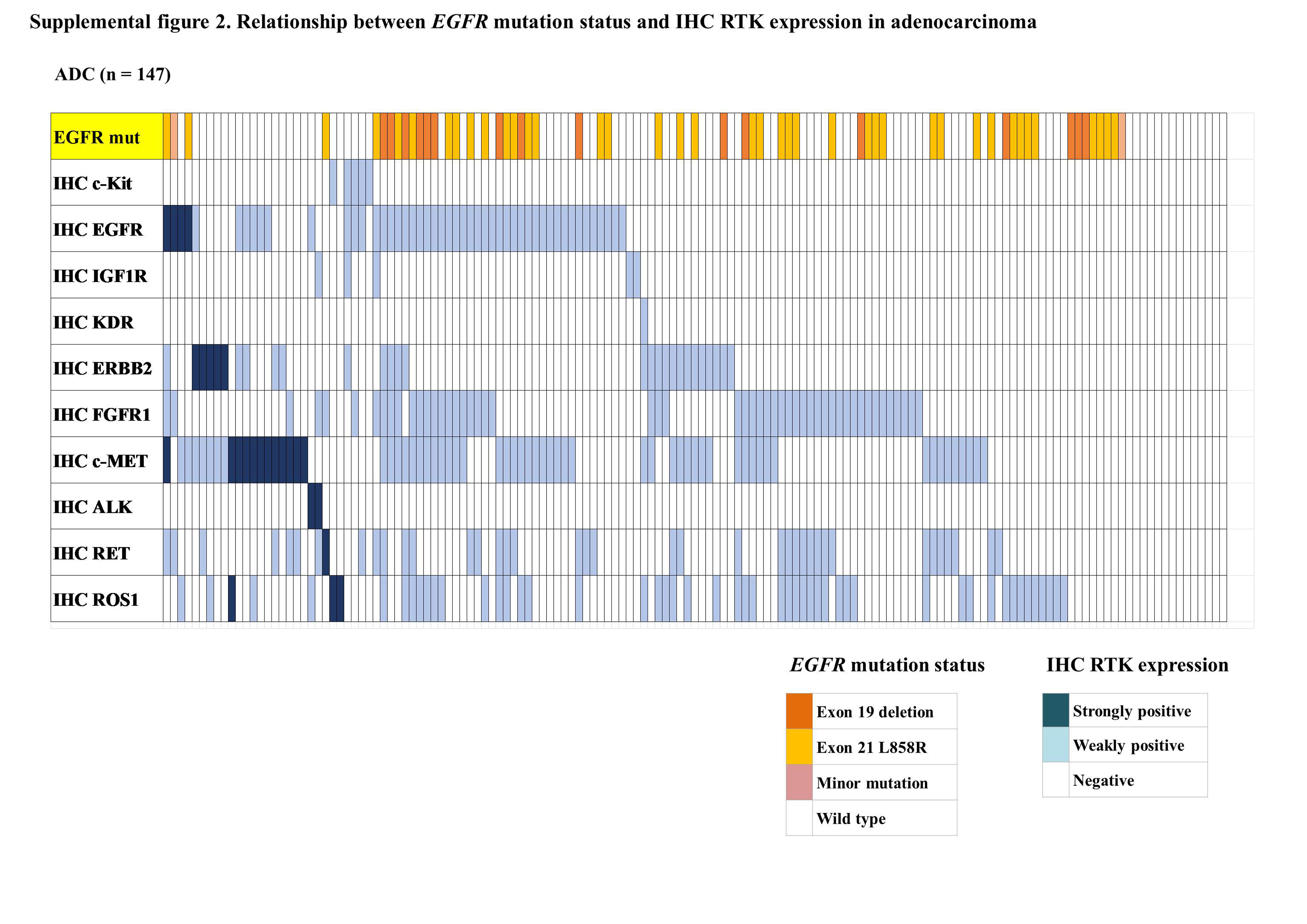

Supplement: Supplementary file 12 — Supplementary material 12 (TIFF 25513 kb) [file 432_2015_1989_MOESM12_ESM.tif]

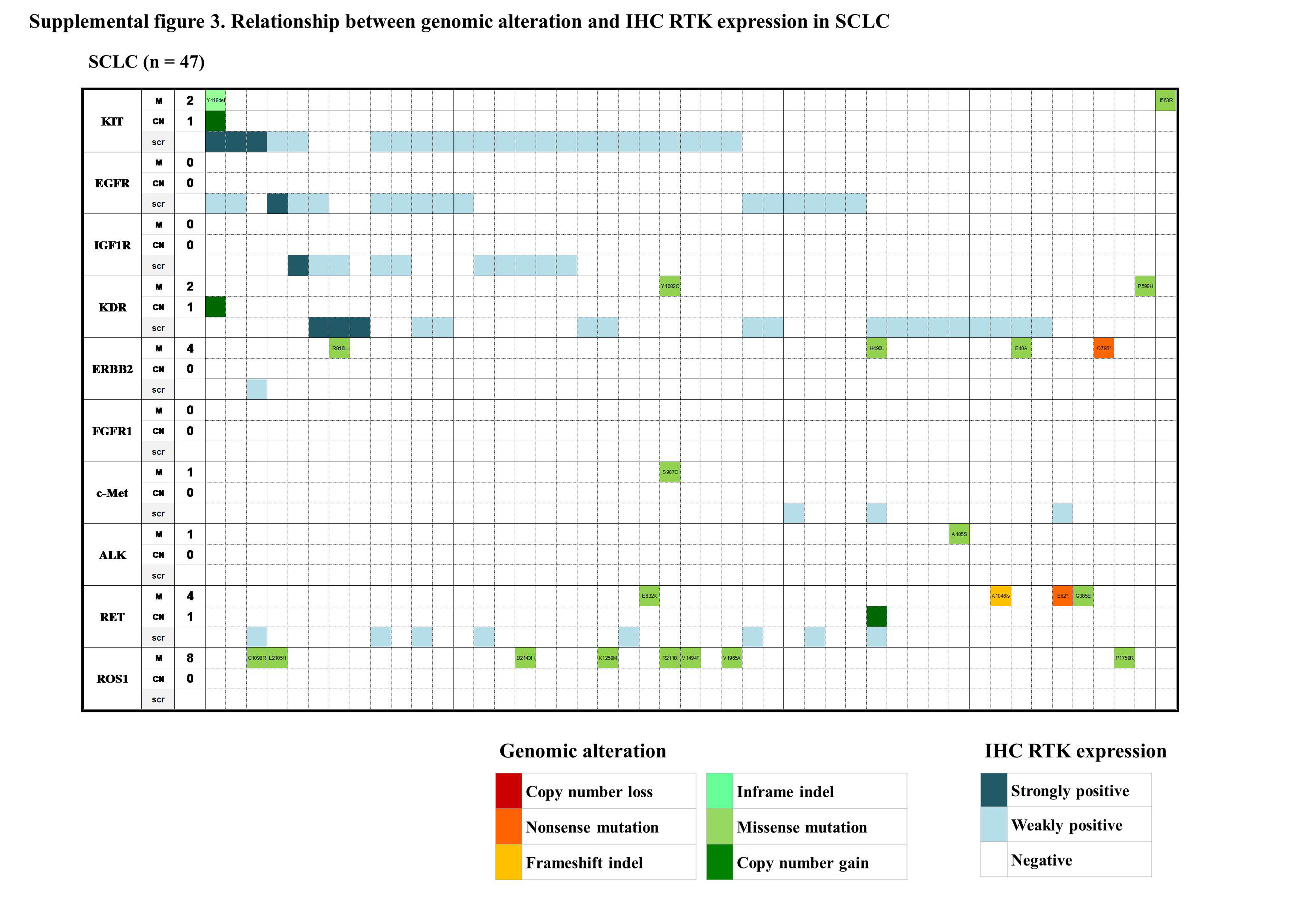

Supplement: Supplementary file 13 — Supplementary material 13 (TIFF 25513 kb) [file 432_2015_1989_MOESM13_ESM.tif]

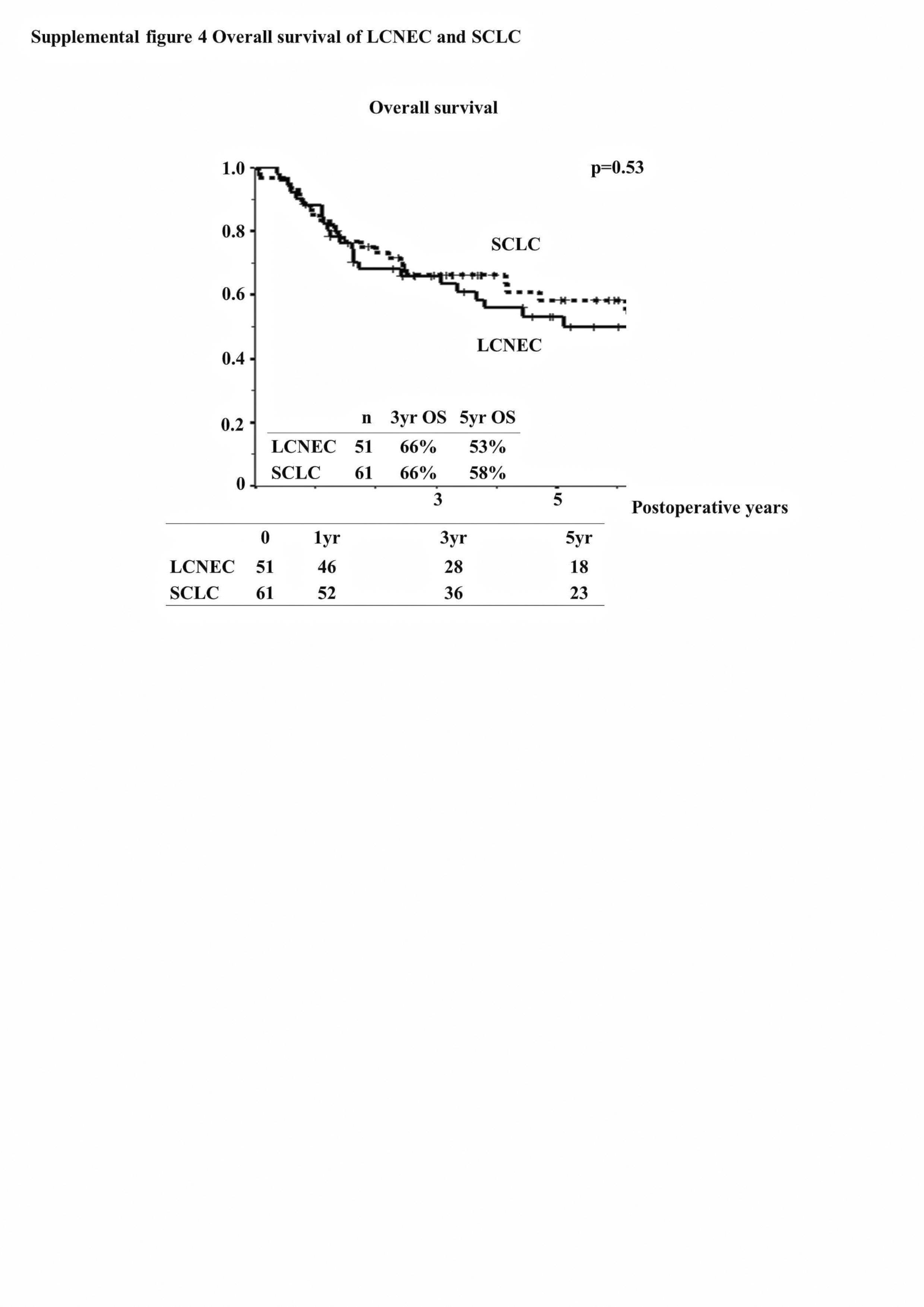

Supplement: Supplementary file 14 — Supplementary material 14 (TIFF 8519 kb) [file 432_2015_1989_MOESM14_ESM.tif]

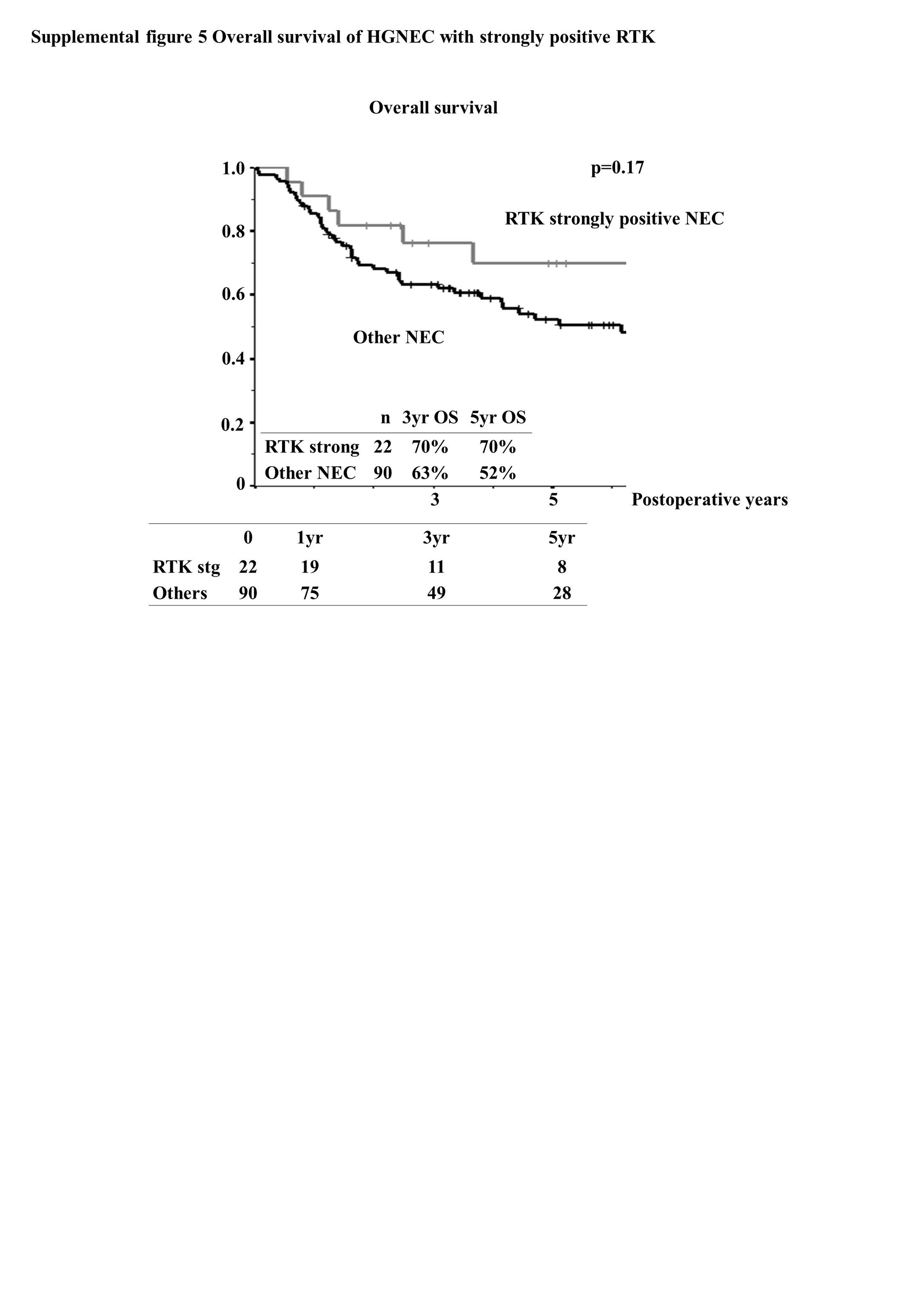

Supplement: Supplementary file 15 — Supplementary material 15 (TIFF 8519 kb) [file 432_2015_1989_MOESM15_ESM.tif]

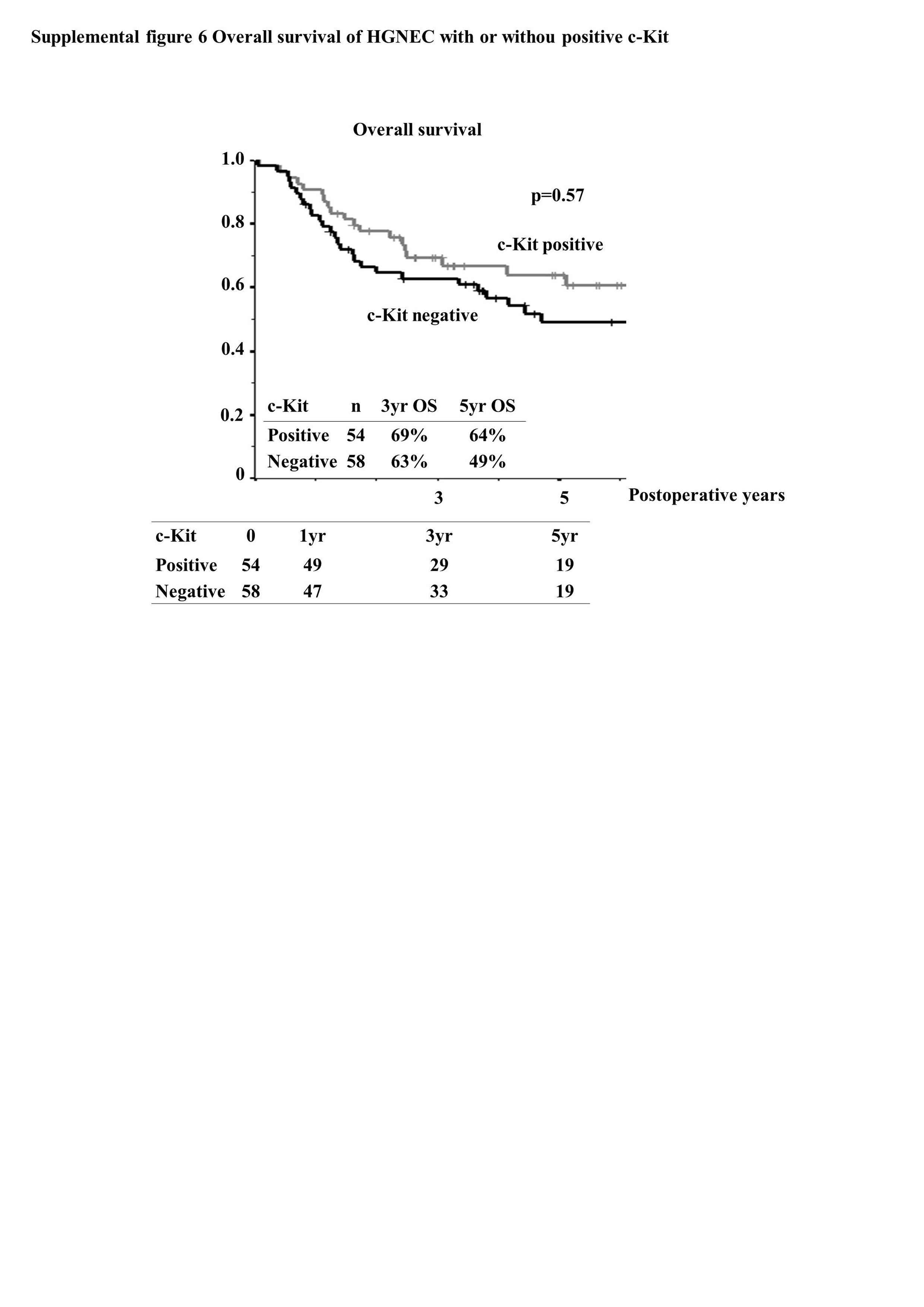

Supplement: Supplementary file 16 — Supplementary material 16 (TIFF 8519 kb) [file 432_2015_1989_MOESM16_ESM.tif]
