# Supplementary material for: Expression profiling of receptor tyrosine kinases in high-grade neuroendocrine carcinoma of the lung: a comparative analysis with adenocarcinoma and squamous cell carcinoma
Source: J Cancer Res Clin Oncol. 2015 May 20;141(12):2159–70. doi: 10.1007/s00432-015-1989-z (PMC4630254; doi:10.1007/s00432-015-1989-z)
Supplement: Supplementary file 17 — Supplementary material 17 (DOCX 33 kb) [file 432_2015_1989_MOESM17_ESM.docx]

**Supplemental table 1 Antibody for immunohistochemical stain to receptor-type tyrosine kinase**

| **RTK** | **origin** | **clone** | **Retrieval** | **Dilution** | **Buffer** | **Company** |
| --- | --- | --- | --- | --- | --- | --- |
| **c-Kit** | rabbit | polyclonal | cooker 99℃, 10min | 1:500 | TRS, pH9 | Dako Cytomation |
| **EGFR** | mouse | H11 | proteinase K | 1:1000 | citrate | Dako Cytomation |
| **IGF1R** | rabbit | polyclonal | cooker 120℃, 4 min | 1:100 | citrate | CST* |
| **KDR** | rabbit | 55B11 | MW, 95℃, 20 min | 1:600 | TRS, pH9 | CST* |
| **ERBB2** | rabbit | 4B5 | 95℃, 64 min** | ** | CC1, pH8.5** | Roche |
| **FGFR1** | rabbit | D8E4 | MW, 95℃, 20 min | 1:500 | citrate | CST* |
| **c-Met** | rabbit | SP44 | 95℃, 64 min** | 1:50 | CC1, pH8.5** | Roche,  Spring bioscience*^3^ |
| **ALK** | mouse | 5A4 | MW, 95℃, 20 min | 1:50 | TRS, pH9 | abcam |
| **RET** | rabbit | EPR2871 | AC, 121℃, 10min | 1:250 | citrate | abcam |
| **ROS1** | rabbit | D4D6 | MW, 95℃, 20 min | 1:100 | TRS, pH9 | CST |

MW, microwave; AC, autoclave; *CST, Cell signaling technology; **: ERBB2 and c-Met were stained in Ventana ultraView pathway in Ventana Benchmark XT stainer (Ventana Medical Systems, Tucson, AZ); *^3^: Antibody for c-Met was purchased from Spring bioscience.

**Supplemental table 2. The number of positive RTK among each histological type**

(A)

| **No. of positive RTK** | **LCNEC**  **n = 51 (%)** | **SCLC**  **n = 61 (%)** | **ADC**  **n = 202 (%)** | **SQCC**  **n = 122 (%)** | **Total** |
| --- | --- | --- | --- | --- | --- |
| **0** | 2 (4) | 8 (13) | 27 (13) | 11 (9) | 48 |
| **1** | 21 (41) | 19 (31) | 44 (22) | 29 (24) | 113 |
| **2** | 15 (29) | 16 (33) | 52 (26) | 51 (42) | 138 |
| **3** | 12 (23) | 6 (18) | 52 (26) | 21 (17) | 96 |
| **4** | 1 (2) | 1 (5) | 20 (10) | 9 (7) | 33 |
| **5** | 0 | 0 | 6 (3) | 1 (1) | 7 |
| **6** | 0 | 0 | 1 (1) | 0 | 1 |

(B)

| **No. of positive RTKs** | **LCNEC**  **n = 51 (%)** | **SCLC**  **n = 61 (%)** | **ADC**  **n = 202 (%)** | **SQCC**  **n = 122 (%)** | **Total** |
| --- | --- | --- | --- | --- | --- |
| **0-1** | 23 (45) | 27 (44) | 71 (35) | 40 (33) | 161 |
| **2 or more** | 28 (55) | 23 (56) | 131 (65) | 82 (67) | 138 |
| **p-value** | 0.93 (vs SCLC) |  |  |  |  |
|  | 0.18 (vs ADC) | 0.20 (vs ADC) |  |  |  |
|  | 0.15 (vs SQCC) | 0.13 (vs SQCC) |  |  |  |

**Supplemental table 3. The number of patients with positive RTKs among each histological type**

(A)

| **RTKs** | **LCNEC**  **n=51 (%)** | **SCLC**  **n=61 (%)** | **ADC**  **n=202 (%)** | **SQCC**  **n=122 (%)** |
| --- | --- | --- | --- | --- |
| **c-Kit** | 25 (49) | 29 (47) | 6 (3) | 5 (4) |
| **EGFR** | 21 (41) | 19 (31) | 63 (31) | 84 (69) |
| **IGF1R** | 16 (31) | 17 (28) | 11 (5) | 76 (62) |
| **KDR** | 15 (29) | 23 (38) | 3 (1) | 37 (30) |
| **ERBB2** | 3 (6) | 1 (2) | 35 (17) | 1 (1) |
| **FGFR1** | 1 (2) | 0 (0) | 71 (35) | 4 (3) |
| **c-Met** | 1 (2) | 4 (6) | 87 (43) | 17 (14) |
| **ALK** | 0 (0) | 0 (0) | 4 (2) | 0 (0) |
| **RET** | 9 (18) | 11 (18) | 59 (29) | 2 (2) |
| **ROS1** | 0 (0) | 0 (0) | 81 (40) | 9 (7) |

(B)

| **p-value** | **LCNEC**  **vs**  **SCLC** | **LCNEC**  **vs**  **ADC** | **LCNEC**  **vs**  **SQCC** | **SCLC**  **vs**  **ADC** | **SCLC**  **vs**  **SQCC** |
| --- | --- | --- | --- | --- | --- |
| **c-Kit** | 0.87 | **<0.01** | **<0.01** | **<0.01** | **<0.01** |
| **EGFR** | 0.27 | 0.18 | **<0.01** | 0.99 | **<0.01** |
| **IGF1R** | 0.69 | **<0.01** | **<0.01** | **<0.01** | **<0.01** |
| **KDR** | 0.35 | **<0.01** | 0.90 | **<0.01** | **0.31** |
| **ERBB2** | 0.22 | **0.04** | 0.05 | **<0.01** | 0.61 |
| **FGFR1** | 0.27 | **<0.01** | 0.63 | **<0.01** | 0.15 |
| **c-Met** | 0.24 | **<0.01** | **0.02** | **<0.01** | 0.14 |
| **ALK** | NA | 0.31 | NA | 0.14 | NA |
| **RET** | 0.95 | 0.09 | **<0.01** | 0.08 | **<0.01** |
| **ROS1** | NA | **<0.01** | 0.05 | **<0.01** | 0.03 |

Mann-Whitney’s U test

**Supplemental table 4 Clinicopathological characteristics of HGNEC due to c-Kit**

|  | **HGNEC** | **HGNEC** | **p-value** |
| --- | --- | --- | --- |
|  | **c-Kit positive** | **c-Kit negative** |  |
|  | **n = 54** | **n = 58** |  |
| **Gender: Men (%)** | 48 (89) | 45 (77) | 0.10 |
| **Age: median (range)** | 68 (42-85) | 67 (22-86) | 0.95 |
| **Smoking: Ever (%)** | 53 (98) | 56 (96) | 0.60 |
| **Pack-year ≥ 50** | 22 (41) | 28 (48) | 0.42 |
| **pStage I** | 34 (63) | 31 (53) |  |
| **II** | 9 (17) | 15 (26) |  |
| **III** | 9 (17) | 12 (21) |  |
| **IV** | 2 (3) | 0 |  |
| **Pleural invasion** | 12 (22) | 27 (46) | <0.01 |
| **Vascular invasion** | 43 (80) | 48 (83) | 0.67 |
| **Lymphatic permeation** | 16 (30) | 25 (43) | 0.14 |
